# Callosal thickness profiles for prognosticating conversion from mild cognitive impairment to Alzheimer’s disease: A classification approach

**DOI:** 10.1002/brb3.1142

**Published:** 2018-11-22

**Authors:** Chris Adamson, Richard Beare, Gareth Ball, Mark Walterfang, Marc Seal, the Alzheimer’s Disease Neuroimaging Initiative

**Affiliations:** ^1^ Developmental Imaging Murdoch Children’s Research Institute Parkville Victoria Australia; ^2^ Department of Medicine Monash University Melbourne Victoria Australia; ^3^ Neuropsychiatry Unit Royal Melbourne Hospital Melbourne Victoria Australia; ^4^ Department of Psychiatry University of Melbourne Melbourne Victoria Australia; ^5^ Florey Institute of Neuroscience and Mental Health Melbourne Victoria Australia

**Keywords:** Alzheimer’s disease, biomarker, classification, corpus callosum, magnetic resonance imaging, segmentation

## Abstract

**Introduction:**

Alzheimer's disease (AD) is the most common form of dementia. Finding biomarkers to prognosticate transition from mild cognitive impairment (MCI) to AD is important to clinical medicine. Promising imaging biomarkers of AD conversion identified so far include atrophy of the cerebral cortex and subcortical gray matter nuclei.

**Methods:**

This study introduces thickness and bending angle of the corpus callosum as a putative white matter marker of MCI to AD conversion. The corpus callosum is computationally less demanding to segment automatically compared to more complicated structures and a subject can be processed in a few minutes. We aimed to demonstrate that callosal shape and thickness measures provide a simple, effective, and accurate prognostication tool in ADNI dataset. Using longitudinal datasets, we classified MCI subjects based on conversion to AD assessed via cognitive testing. We evaluated the classification accuracy of callosal shape features in comparison with the existing “gold standard” cortical thickness and subcortical gray matter volume measures.

**Results:**

The callosal thickness measures were less accurate in classifying conversion status by cognitive scores compared to gray matter measures for AD.

**Conclusions:**

While this paper presented a negative result, this method may be more suitable for a disease of the white matter.

## 1 INTRODUCTION

Alzheimer's disease (AD) is the most common form of dementia (Weiner et al., 2013). The neuropathology of AD is hypothesized to be a cascade of β‐amyloid plaques, tau‐mediated neuronal injury, and tissue loss leading to cognitive impairments (Jack et al., 2010). The putative course of clinical progression in AD begins with mild cognitive impairment (MCI), later converting to AD as cognitive abilities decline.

Clinically, the level of cognitive impairment is typically established using tests of mental status including the Clinical Dementia Rating (CDR) and Mini Mental State Exam (MMSE). However, the development of robust imaging biomarkers for AD has the potential for clinical impact (Frisoni, Fox, Jack, Scheltens, & Thompson, 2010), particularly in prognostication of cognitive decline. More specifically, an important goal for neuroimaging biomarkers is to accurately predict whether a patient presenting with mild cognitive impairment (MCI) initially will degrade further to severe cognitive impairment, converting to AD, or remain stable.

To aid the search for imaging biomarkers of AD, the ADNI project was conceived to provide a longitudinal, publicly available dataset for researchers (Jack et al., 2008). A major focus of ADNI‐based imaging studies has been to investigate whether the trajectories of cortical and subcortical gray matter atrophy predict cognitive decline (Weiner et al., 2013). Cortical gray matter atrophy occurs along a temporo‐spatial gradient with disease progression, occurring first in the temporal cortex and followed by occipital, parietal, frontal atrophy (Fennema‐Notestine et al., 2009). In patients with AD, gray matter volume is significantly reduced across the whole cortex, apart from the primary motor/sensory and visual cortex which are relatively spared. Examination of volumetric and shape trajectory with disease progression in the subcortical gray matter nuclei and ventricles has shown marked atrophy of the hippocampus, amygdala, and ventricular enlargement in AD (Fennema‐Notestine et al., 2009; Qiu, Fennema‐Notestine, Dale, & Miller, 2009). Willette, Calhoun, Egan, and Kapogiannis (2014) applied independent component analysis to gray matter tissue maps to achieve approximately 80% accuracy for classifying MCI converters versus MCI nonconverters.

Relatively few papers have examined white matter atrophy in AD (Migliaccio et al., 2012; Zhang et al., 2009). Migliaccio et al. (2012) compared healthy controls to AD patients (non‐ADNI) and found atrophy in lateral temporal and parietal regions, including cingulum and posterior corpus callosum. Zhang et al. (2009) examined diffusion metrics to characterize white matter microstructure in Alzheimer's disease compared to front‐temporal dementia. This study found that white matter microstructure was less affected in AD compared to frontotemporal dementia.

Typically, these studies rely on high‐dimensional, nonlinear image registration techniques (Davatzikos, Genc, Xu, & Resnick, 2001; Shen & Davatzikos, 2003) and/or complex cortical segmentation procedures (Fischl, 2012) that can be computationally costly when applied to large cohorts and may require manual intervention or editing in extreme anatomical cases. Additionally, these studies typically used voxel‐based morphometry, which is sensitive to chosen parameters such as smoothing size.

We recently introduced a method for extracting the midsagittal plane, corpus callosum and generating thickness profiles (Adamson et al., 2011; Adamson, Beare, Walterfang, & Seal, 2014). This process can be quickly applied to any T1‐weighted MR image to summarize callosal curvature and midsagittal callosal thickness within 8 subdivisions. Midsagittal callosal area closely correlates with total myelinated axonal fiber count (Riise & Pakkenberg, 2011), and studies show greater thickness of the corpus callosum is linked to measures of general cognitive ability (Luders et al., 2007). To date, there has been one other study of CC morphology change with disease progression in ADNI (Elahi, Bachman, Lee, Sidtis, & Ardekani, 2015). This paper used regional area and circularity, a measure of bending, and showed statistically significant differences between MCI converters and nonconverters. However, these group differences were not prognostic in nature. We propose that callosal measures serve as a surrogate marker of cerebral atrophy, providing an alternative to computationally taxing whole‐brain approaches. The aim of this paper was to test whether callosal thickness is as effective in classifying conversion from MCI to AD from first‐visit data as more comprehensive measures of cerebral atrophy.

## 2 METHODS

Data used in the preparation of this article were obtained from the Alzheimer's Disease Neuroimaging Initiative (ADNI) database (adni.loni.usc.edu). The ADNI was launched in 2003 as a public–private partnership, led by Principal Investigator Michael W. Weiner, MD. The primary goal of ADNI has been to test whether serial magnetic resonance imaging (MRI), positron emission tomography (PET), other biological markers, and clinical and neuropsychological assessment can be combined to measure the progression of mild cognitive impairment (MCI) and early Alzheimer's disease (AD). For up‐to‐date information, see www.adni-info.org. Longitudinal imaging data from a total of 556 subjects (age 55.75–92.66) were downloaded. The imaging schedule involved one or two initial screening sessions followed by yearly follow‐up scans; the number of follow‐up scans varied between subjects.

Subjects were initially grouped per cognitive ability into Alzheimer's disease (AD), mild cognitive impairment (MCI), and otherwise healthy controls (CTL). Cognitive ability was assessed using the global score on the Clinical Dementia Rating, which was administered at every visit. The CDR score has the following possible values (0 = none, 0.5 = questionable, 1 = mild, 2 = moderate, 3 = severe). Grouping criteria were as follows: AD—scores of CDR ≥ 1 for all visits, MCI—initial visit score of CDR = 0.5, CTL—scores of CDR = 0 for all visits. Demographic information for these groups is presented in Table 1.

**Table 1:** Demographic information of subject groups according to classification into Alzheimer's disease (AD), mild cognitive impairment (MCI), and healthy controls (CTL)

	AD	MCI	CTL	Total
# images	437	1606	529	2,572
# subjects	136	285	135	556
Age (years) mean (*SD*)	75.70 (7.59)	75.99 (7.34)	77.03 (7.44)	75.14 (7.08)
Males	68	101	66	235

## 3 NEUROIMAGING DATA PREPROCESSING

### 3.1 Callosal thickness

Callosal thickness measurements were obtained with an automated callosal thickness profile generation pipeline (Adamson et al., 2014). Briefly, this extracts the midsagittal slice, segments the corpus callosum, and generates cross‐sectional traversals along the midline of the corpus callosum; the arc lengths of these traversals define callosal thickness. Mean thickness was calculated within 8 callosal subdivision defined by the parcellation schemes of Witelson (1989) and Hofer and Frahm (2006). Additionally, a callosal bending angle was computed as the angle between vectors emanating from the midpoint of the corpus callosum to the apices of the genu and splenium.

### 3.2 Subcortical volumes

Deep gray matter structure volumes were extracted using FIRST (Patenaude, Smith, Kennedy, & Jenkinson, 2011). Images were initially preprocessed using SPM12 “new segment” from which WM, GM, and CSF tissue probability maps and bias‐corrected images were obtained. Bias‐corrected images were processed by Patenaude et al. (2011; FSL 5.0.9) using default options. Volumes of the following structures were obtained from segmentation in both left and right hemispheres: hippocampus, amygdala, accumbens, putamen, pallidum, thalamus, and caudate.

### 3.3 Intracranial volume

Intracranial volume was estimated as the sum of the WM, GM, and CSF volumes; SPM ICV estimates were previously shown to correlate closely to ground truth (Weiner et al., 2013). Callosal thickness measure and deep gray nuclei volumes were normalized by intracranial volume.

### 3.4 Cortical thickness

Cortical thickness estimates were obtained using Freesurfer 5.3.0 (Fischl, 2012). Anatomical localization into 34 regions per hemisphere was performed using the Desikan–Killiany atlas (Desikan et al., 2006), and measures of mean cortical thickness were extracted for each region in both left and right hemispheres.

### 3.5 Feature sets

Several aggregate feature sets were formed from the derived imaging measures for comparison. These included callosal features (CC, *n* = 9); deep gray volumes (FIRST, *n* = 14); a joint set of callosal features and deep nuclear volumes (CCFIRST, *n* = 23); regional cortical thickness estimates from Freesurfer (FS, *n* = 68); cortical thickness and deep gray volumes (FSFIRST, *n* = 82); and all measures (*n* = 91).

### 3.6 Adjustment for healthy aging

As a final preprocessing step, all feature values were adjusted to account for effects of neurotypical aging. A line of best fit was computed for each feature against age using only the healthy control data. The data used for classification were the residuals to these lines of best fit.

### 3.7 Classification

Linear support vector machines, as implemented in scikit‐learn (LinearSVC; 0.18.1; Fan, Chang, Hsieh, Wang, & Lin, 2008; Pedregosa et al., 2011), were used for classification training. Feature selection and parameter tuning were performed using threefold nested, stratified cross‐validation within each training fold. Feature selection was performed using the margin‐maximizing feature elimination method (MFE; Aksu, Miller, Kesidis, & Yang, 2010). LinearSVC is dependent upon a regularization term (C) which weighs the contribution from the data fidelity term. A grid search was used to select C from {10^−7^, 10^−6^, …, 10^2^}. The classifier was then trained using the selected features and optimized parameter choice on the full training set. Feature importance was assessed by calculating the proportion of folds in which a feature was selected.

In this paper, the definition of conversion to AD is based on cognitive decline and on CDR scores, which have been used as gold standard labels in a traditional supervised classification problem. Classification accuracy was determined by the number of subjects correctly identified as converting from MCI to AD based on CDR test scores indicated mild, moderate, or severe dementia (CDR ≥ 1) on one or more visits. Nonconvertors scored CDR = 0.5 for all visits. The MCI converters are labeled C‐CDR, and the MCI nonconverters are labeled N‐CDR.

We estimate generalizability using the following classification experiments: CTL versus AD and C‐CDR versus N‐CDR. Classifier generalizability refers to the labeling accuracy of the classifier on unseen test data, here unseen means data not used in classifier training. Here, classifier generalizability of a given imaging feature set (CC; FIRST; CCFIRST; FS; FSFIRST; ALL) was estimated using cross‐validation on 100 random 80%/20% train/test splits. Generalization accuracy rates were compared between each pair of feature sets statistically via 2‐sample *t* tests. Distributions of generalization accuracy were found to be close to Gaussian using quantile–quantile plots (data not shown), and thus, *t *tests were appropriate.

## 4 RESULTS

### 4.1 Generalizability tests

**Figure 1:**
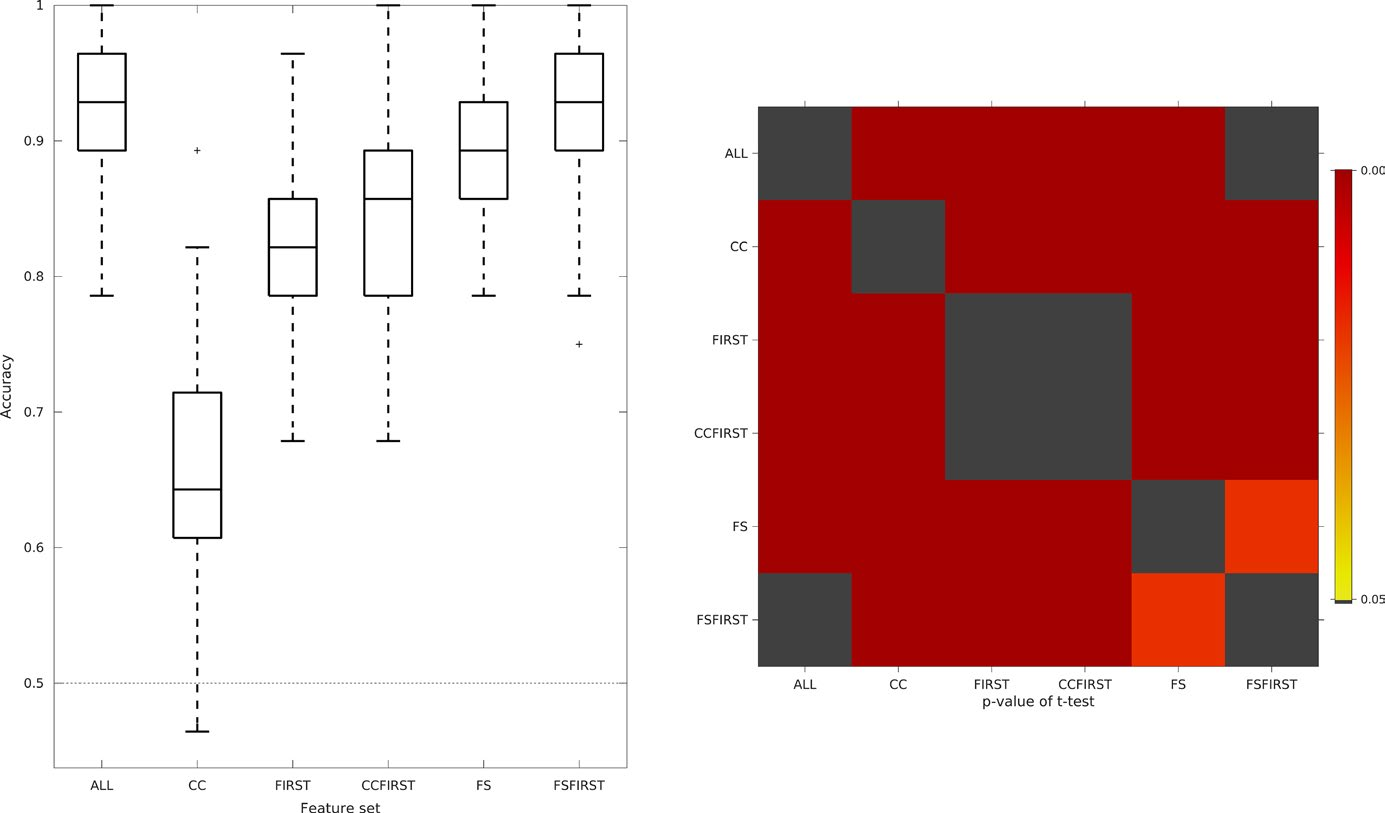
Box plots of cross‐validated generalizability rates for classification of CTL/AD for all feature sets using CTL/AD groups as training data. Chance‐level and noninformative accuracy is denoted by the line at 0.5. *p*‐values for pairwise *t* tests are shown in the inset

Figure 1 shows cross‐validated classification accuracy rates for CTL/AD subjects for all feature sets based on 100 80%/20% train/test stratified splits.

Generalization accuracy for the singular feature sets was ranked, in increasing order, as follows: CC, FIRST, FS. Aggregating feature sets showed that adding the CC features to FIRST, and FIRST to FS yielded marginal improvements. Using all feature sets gives equivalent accuracy to FSFIRST. Supporting information Figures S1–S6 show the feature selection probabilities using MFE across cross‐validation splits for all feature sets. The most informative features per set were entorhinal and middle temporal cortical thickness, hippocampal volume (ALL and FSFIRST), hippocampal volume, amygdala, anterior midbody and splenium (CCFIRST), entorhinal cortex, middle temporal, parahippocampal (FS). In the CC feature set, all features except for genu and bending angle were selected with high probability.

**Figure 2:**
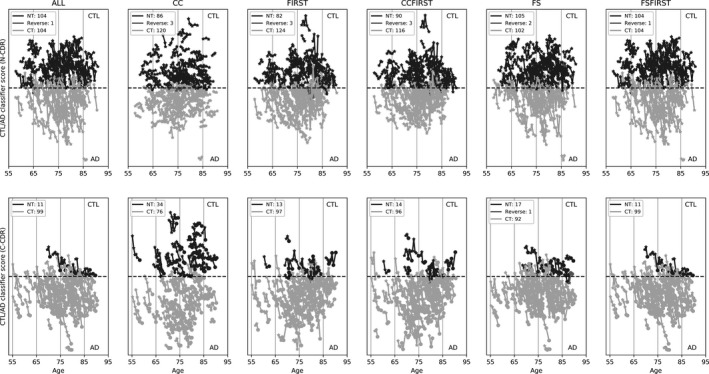
Classification scores for MCI patients using the classifier trained on CTL/AD for each feature set. The top row shows nonconverting (N‐CDR) patients, and the bottom row shows converting MCI patients (C‐CDR). In each plot, light gray represents MCI patients who converted based on brain trajectory (CT), and dark gray denotes those that did not (NT). The group “Reverse” (mid‐gray) denotes subjects that unexpectedly transitioned from AD to CTL. The star markers denote visits of CDR = 0.5, and the circle markers denote CDR ≥ 1

Figure 2 shows the CTL/AD classification decision scores for all feature sets for MCI converters (C‐CDR) and nonconverters (N‐CDR). Each connected line denotes CTL/AD classifier scores across all visits for each subject. Small proportions of subjects unexpectedly transitioned from AD to CTL. The proportions of CTL versus AD classified patients show that the nonconverters are almost equally classified CTL or AD, while the converters are more often classified AD than CTL.

### 4.2 Classification of MCI to AD conversion

**Figure 3:**
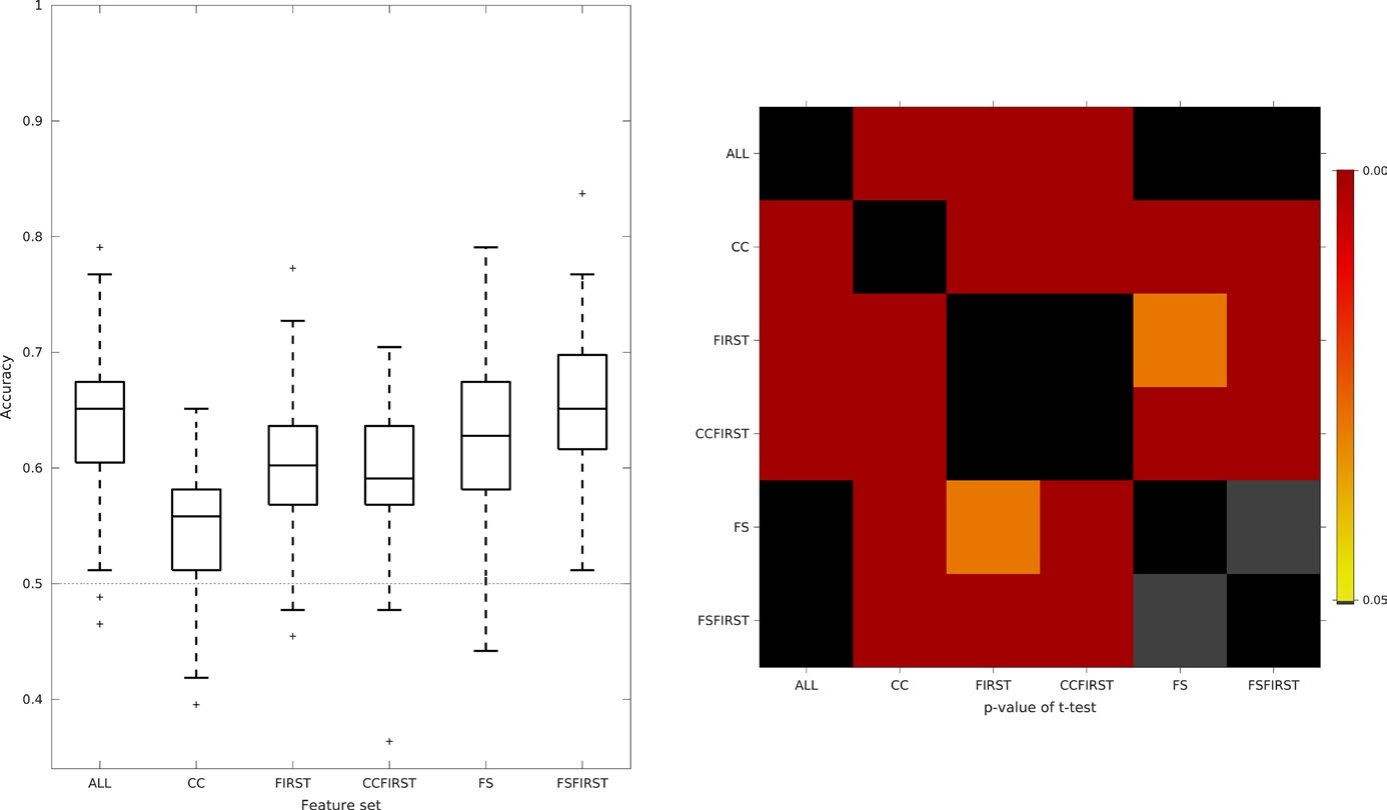
Box plots of cross‐validated generalizability rates for the N‐CDR/C‐CDR classification experiment for all feature sets. Chance‐level and noninformative accuracy is denoted by the line at 0.5. *p*‐values for pairwise *t* tests are shown in the inset

We tested the ability of each feature set to classify nonconverting and converting MCI patients. Classification results per feature set are shown in Figure 3. Classification accuracy mirrors that in the CTL/AD scenario (Figure 1). The singular feature set generalization accuracies were ordered as follows: CC < FIRST < FS. FSFIRST and ALL feature sets gave the highest scores. FIRST and CCFIRST gave intermediate scores. Feature selection probabilities are illustrated in Supporting information Figures S7–S12. The most informative features were: entorhinal cortex, middle temporal, inferior parietal, hippocampal volume (ALL and FSFIRST; Supporting information Figures S7 and S12), all regions except for genu (CC; Supporting information Figure S8), hippocampus, right accumbens, left putamen, anterior midbody of the CC (CCFIRST; Supporting information Figure S4). In the FIRST feature set, the selection probability rankings remain largely the same with hippocampal volumes being the most informative features although the probabilities do not vary greatly across structure suggesting few uniquely key features. Similarly, all features in the CC feature set were selected with high probability.

## 5 DISCUSSION

In this paper, we presented a classification framework for prognostication of conversion to AD from MCI using brain volume patterns. This paper examines whether callosal thickness profiles could be an imaging biomarker for prognosticating conversion from MCI to AD. Results showed that callosal thickness profiles did not achieve comparable accuracy to existing gray matter based “gold standards.”.

Early classification approaches applied to the ADNI dataset attempted to separate subjects using the by‐CDR approach used in this paper (Filipovych & Davatzikos, 2011; Misra, Fan, & Davatzikos, 2009). In these papers, voxel‐wise measures of gray matter expansion or contraction required to warp to a common template were used as feature sets. Feature selection identified temporal cortical gray matter volume and hippocampal volume as being informative for classification. Classification accuracies for the CTL/AD stage were high at 94% (Misra et al., 2009) and 80% (Filipovych & Davatzikos, 2011). However, the N‐CDR/C‐CDR classification accuracy rates were, on average, lower. In Ref. (Filipovych & Davatzikos, 2011), the classification accuracy rates according to converter status were C‐CDR 79.4%, N‐CDR 51.7%. In Ref. (Misra et al., 2009), cross‐validation accuracy rates ranged between 75% and 80%. The issue addressed by Aksu, Miller, Kesidis, Bigler, and Yang (2011) was the use of the CDR as the sole definition of MCI to AD conversion. Predicting CDR values, and thus conversion, from brain markers carries uncertainties of structure/function relationships with the added variance of high variability of cognitive testing scores (Chou et al., 2010).

This paper compared callosal, cortical, and subcortical gray matter volumetric feature sets for CTL/AD and MCI converter classification. Accuracy was highest when classifying control and AD patients using cortical thickness features derived from Freesurfer (90%), followed by FIRST‐derived deep gray volumes (82%) and callosal thickness measurements (63%). Feature selection probabilities indicated that the most informative features were left and right entorhinal cortical thickness and hippocampal volumes. Atrophy of these structures is commonly reported in early disease states (Weiner et al., 2013). There were no specific regions of the CC that were particularly informative and thus suggestive of a global CC atrophy occurring in AD (Ardekani, Bachman, Figarsky, & Sidtis, 2014; Teipel et al., 2002). The review of Di Paola, Spalletta, and Caltagirone (2010) of callosal atrophy work found the most robust findings were atrophy of genu and splenium with varying results in the midbody. The finding of posterior callosal atrophy of Migliaccio et al. (2012) was not found; however, the feature selection probabilities were computed in a different fashion to the *p*‐value based results in that paper. The lower accuracy of the CC suggests that AD is a disease affecting gray matter more than white matter. The white matter atrophy reported by Migliaccio et al. (2012) was confined to the posterior corpus callosum and its nearby cortical projections. The spatial extent of these findings was sparse compared to the brain‐wide atrophy of cortical gray matter and hippocampal tissue seen in other AD research (Weiner et al., 2013). This paper performed comparison of all feature sets under the by‐CDR conversion definition. The classification accuracies of all feature sets were relatively poor with averages ranging from 60% to 70%; this agrees with previous work (Aksu et al., 2011). The CC feature set was particularly poor with an average accuracy rate of 56%.

Feature selection probabilities were computed to assess which brain structures were the most informative in prognosticating disease state. Cortical thickness, particularly temporal and parietal cortex, and hippocampal volumes were consistently highest ranked for the CTL/AD classification scenario; these findings agree with earlier work (Risacher et al., 2010; Weiner et al., 2013). In addition to these brain structures inferior parietal cortex, amygdala and posterior CC became more highly ranked. The putative progression of AD in terms of cortical atrophy takes on the following trajectory: temporal, occipital, parietal, frontal with relative sparing of primary visual and motor cortex. The cortical projections from the posterior CC are the temporal, occipital, and parietal cortices (Hofer & Frahm, 2006). Thus, the brain structures that are more informative for distinguishing subjects per functional impairment are associated with later stage atrophy. This finding also agrees with previous work (Aksu et al., 2011; Di Paola et al., 2010; Teipel et al., 2002).

The generalization accuracy rates presented in this paper for gray matter structures at around 60%–70% are inferior to those achieved in other works on ADNI (Misra et al.^2009^; Willette et al., 2014). However, the goal of this paper was to only to compare feature set generalization ability for a single classification algorithm.

Other methods have modeled the CC as a 3D object by incorporating the midsagittal and parasagittal slices and modeling thickness using medial axes (Styner, Gerig, Lieberman, Jones, & Weinberger, 2003). Our study focused on a 2D, single‐slice shape representation to make a shape representation to be as compact as possible. Additionally, extending the segmentation to include parasagittal slices should not add a great deal of information not already captured in the midsagittal slice.

## 6 CONCLUSION

This paper assessed accuracy of prediction of conversion from MCI to AD using first‐visit brain volume measures. We investigated whether the thickness of the midsagittal corpus callosum and its bending angle could be used as a biomarker for AD conversion compared to existing candidate biomarkers: cortical thickness and subcortical gray nuclei volumes. The CC was less accurate in predicting MCI to AD conversion. However, previous work suggests that the AD setting may not be optimal for the presented method, but it is worthwhile for diseases of white matter. We propose that callosal measures represent a quick, simple addition to the search for an imaging biomarker in AD. Future research may incorporate these measures to aid clinical assessments in a rapid fashion.

## CONFLICT OF INTEREST

None declared.
