# Supplementary material for: Callosal thickness profiles for prognosticating conversion from mild cognitive impairment to Alzheimer’s disease: A classification approach
Source: Brain Behav. 2018 Nov 22;8(12):e01142. doi: 10.1002/brb3.1142 (PMC6305917; doi:10.1002/brb3.1142)
Supplement: Supplementary file 1 [file BRB3-8-e01142-s001.docx]

# Supplementary Figures

## Titles

Figure S1: Feature selection probabilities for the CTL/AD cross-validation procedure for the “ALL” feature set.

Figure S2: Feature selection probabilities for the CTL/AD cross-validation procedure for the “CC” feature set.

Figure S3: Feature selection probabilities for the CTL/AD cross-validation procedure for the “FIRST” feature set.

Figure S4: Feature selection probabilities for the CTL/AD cross-validation procedure for the “CCFIRST” feature set.

Figure S5: Feature selection probabilities for the CTL/AD cross-validation procedure for the “FS” feature set.

Figure S6: Feature selection probabilities for the CTL/AD cross-validation procedure for the “FSFIRST” feature set.

Figure S7: Feature selection probabilities per the cross-validation procedure, by-CDR classification scenario, “ALL” feature set.

Figure S8: Feature selection probabilities per the cross-validation procedure, by-CDR classification scenario, “CC” feature set.

Figure S9: Feature selection probabilities per the cross-validation procedure, by-CDR classification scenario, “FIRST” feature set.

Figure S10: Feature selection probabilities per the cross-validation procedure, by-CDR classification scenario, “CCFIRST” feature set.

Figure S11: Feature selection probabilities per the cross-validation procedure, by-CDR classification scenario, “FS” feature set.

Figure S12: Feature selection probabilities per the cross-validation procedure, by-CDR classification scenario, “FSFIRST” feature set.

Figure S13: Feature selection probabilities per the cross-validation procedure, by-Trajectory classification scenario, “ALL” feature set.

Figure S14: Feature selection probabilities per the cross-validation procedure, by-Trajectory classification scenario, “CC” feature set.

Figure S15: Feature selection probabilities per the cross-validation procedure, by-Trajectory classification scenario, “FIRST” feature set.

Figure S16: Feature selection probabilities per the cross-validation procedure, by-Trajectory classification scenario, “CCFIRST” feature set.

Figure S17: Feature selection probabilities per the cross-validation procedure, by-Trajectory classification scenario, “FS” feature set.

Figure S18: Feature selection probabilities per the cross-validation procedure, by-Trajectory classification scenario, “FSFIRST” feature set.


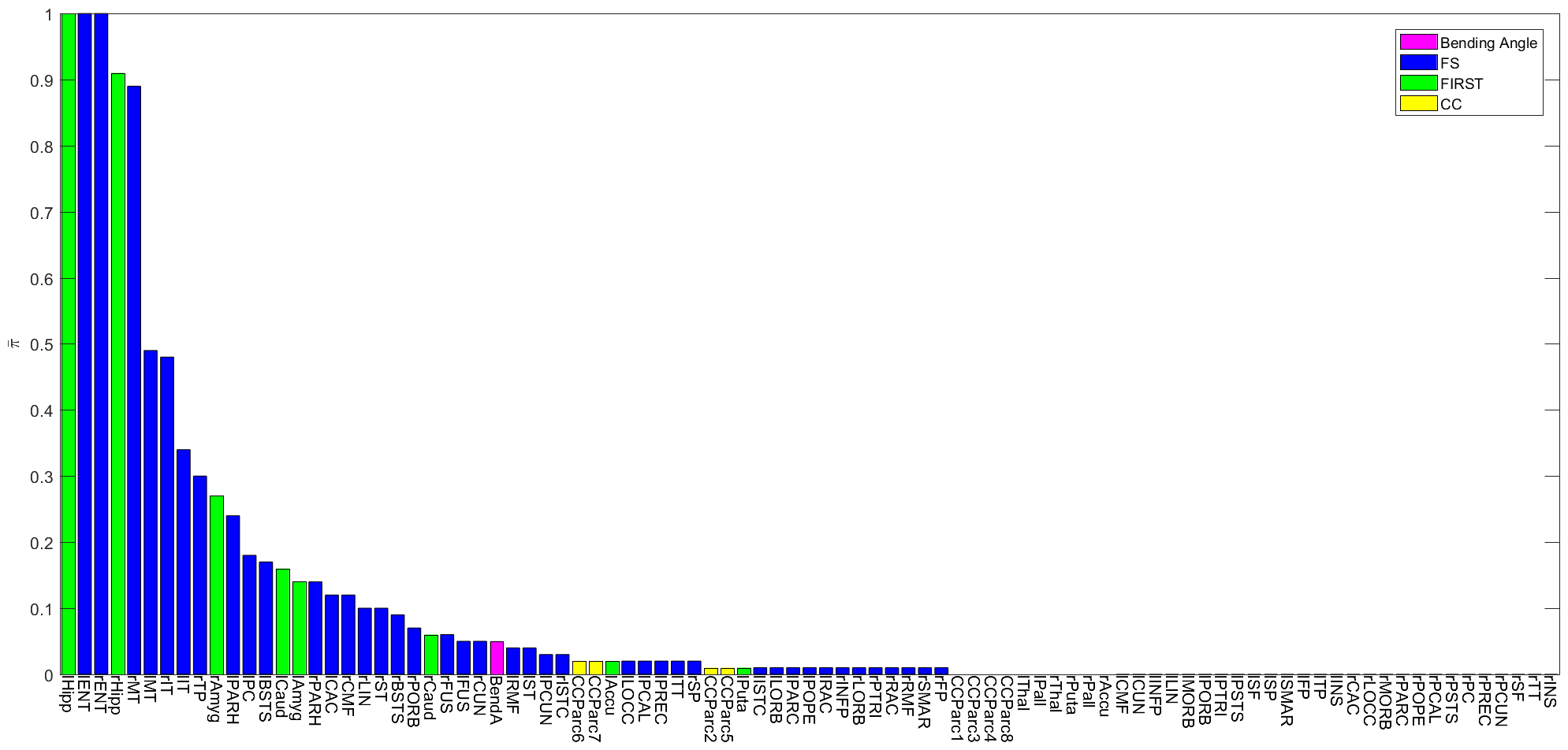

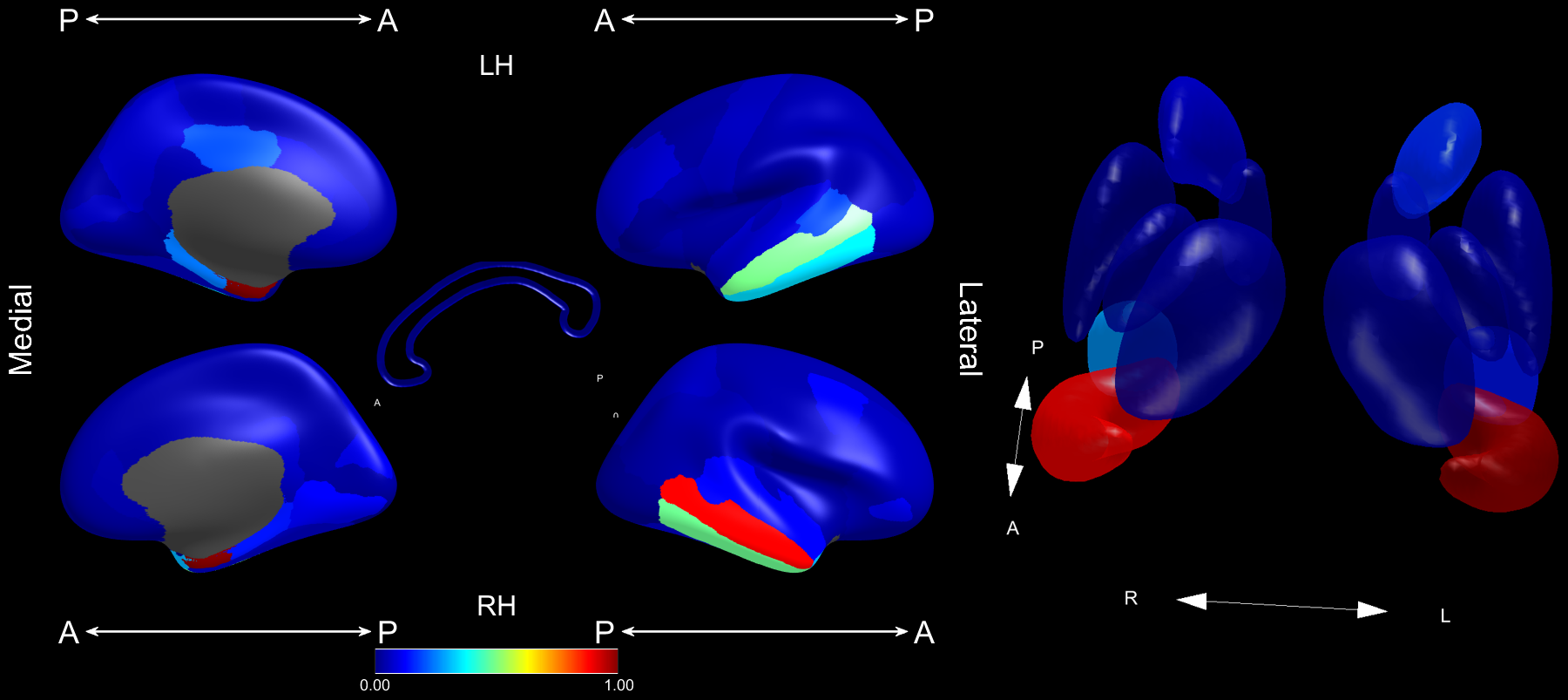


Figure S1: Feature selection probabilities for the CTL/AD cross-validation procedure for the “ALL” feature set. Upper panel: sorted feature selection probabilities, coloured by source feature set. Lower panel: cortical thickness and regional callosal thickness (left) and subcortical grey matter nuclei (right).


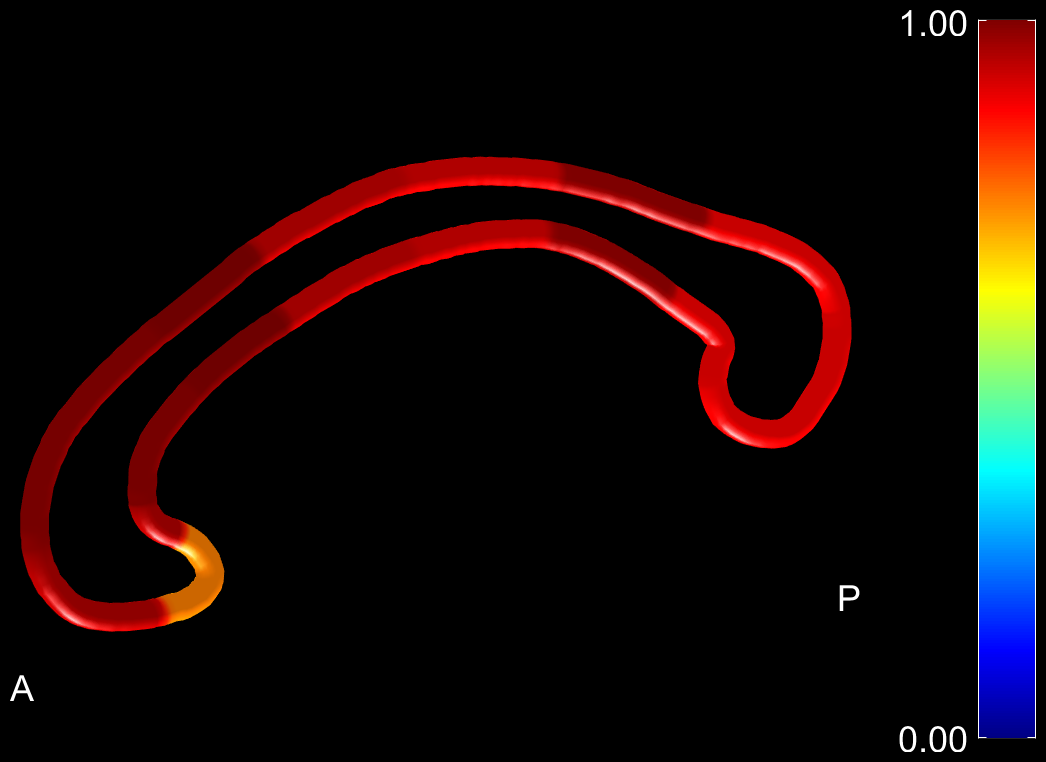

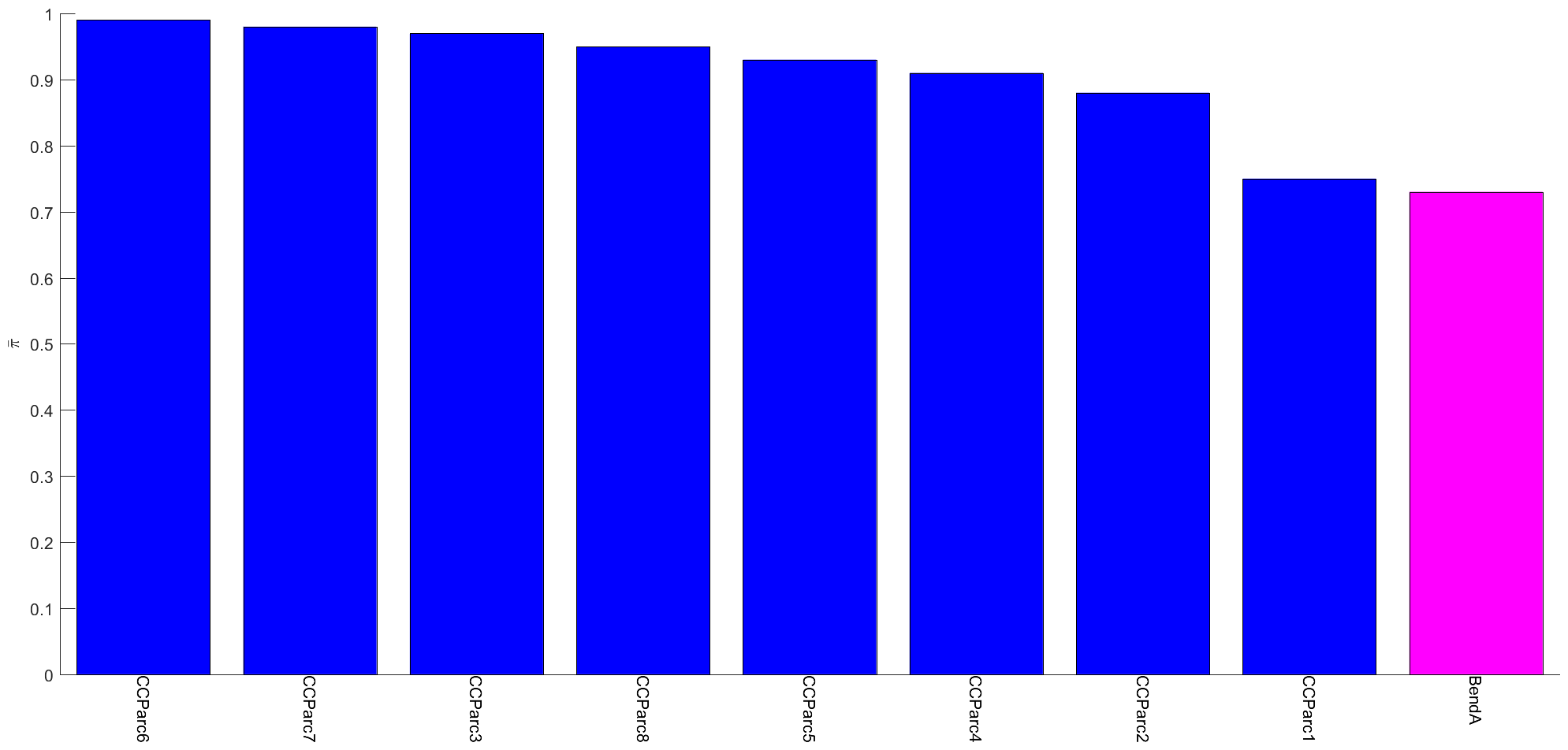


Figure S2: Feature selection probabilities for the CTL/AD cross-validation procedure for the “CC” feature set. Upper panel: sorted feature selection probabilities. Lower panel: Regional callosal thickness.


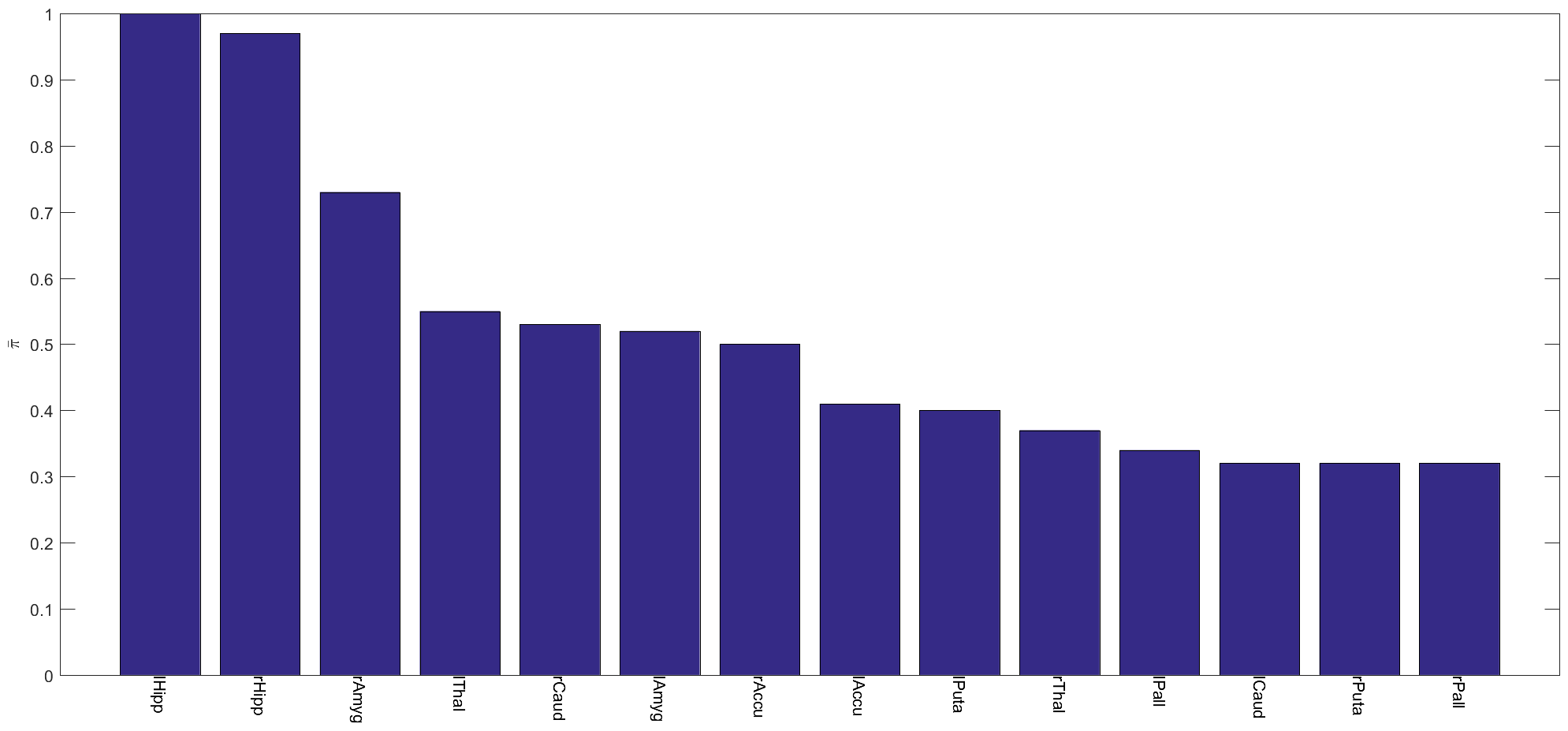

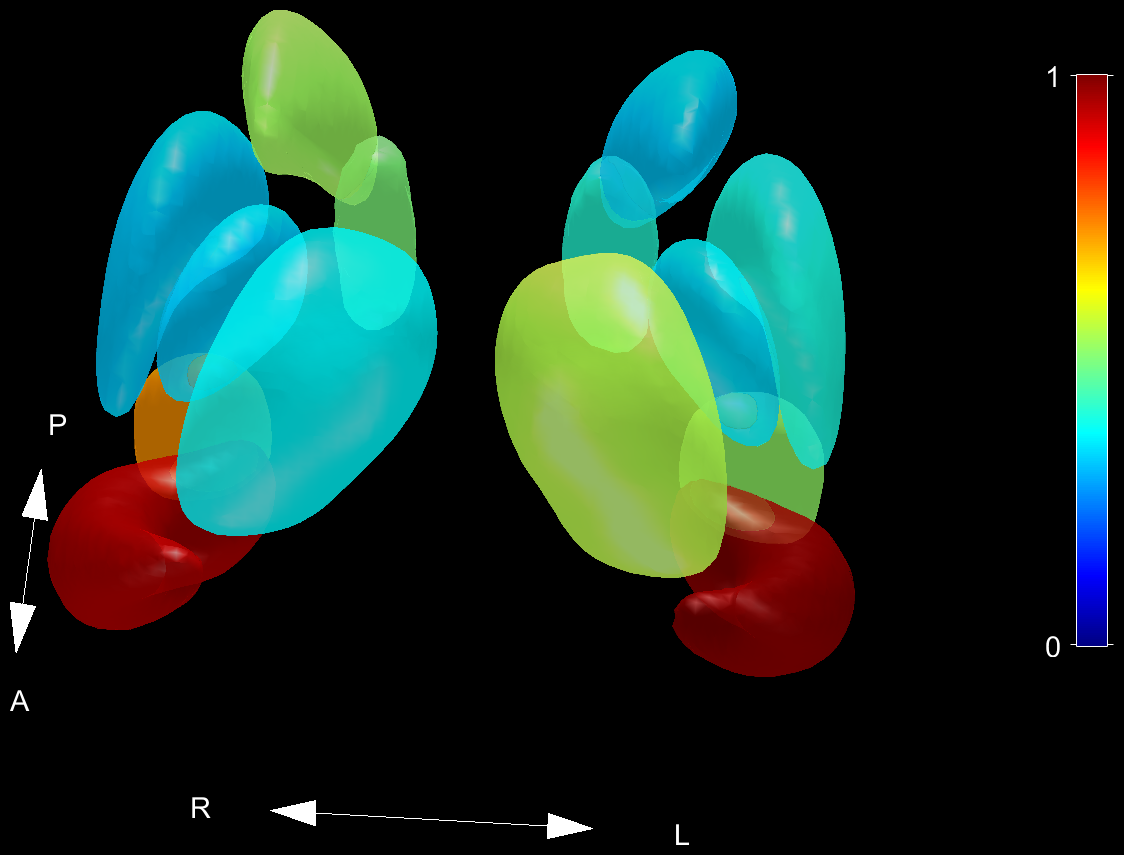


Figure S3: Feature selection probabilities for the CTL/AD cross-validation procedure for the “FIRST” feature set. Upper panel: sorted feature selection probabilities, coloured by source feature set. Lower panel: subcortical grey matter nuclei.


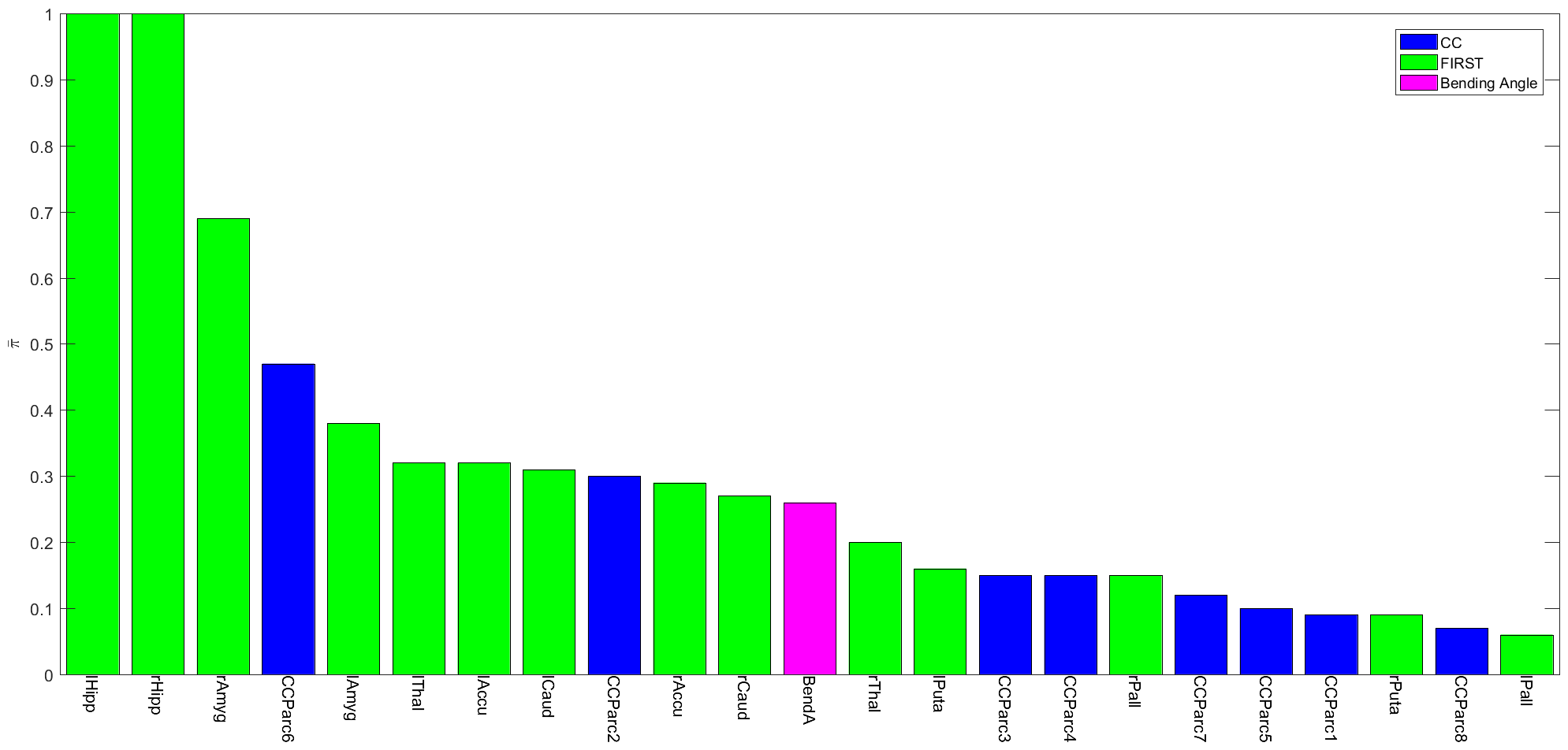

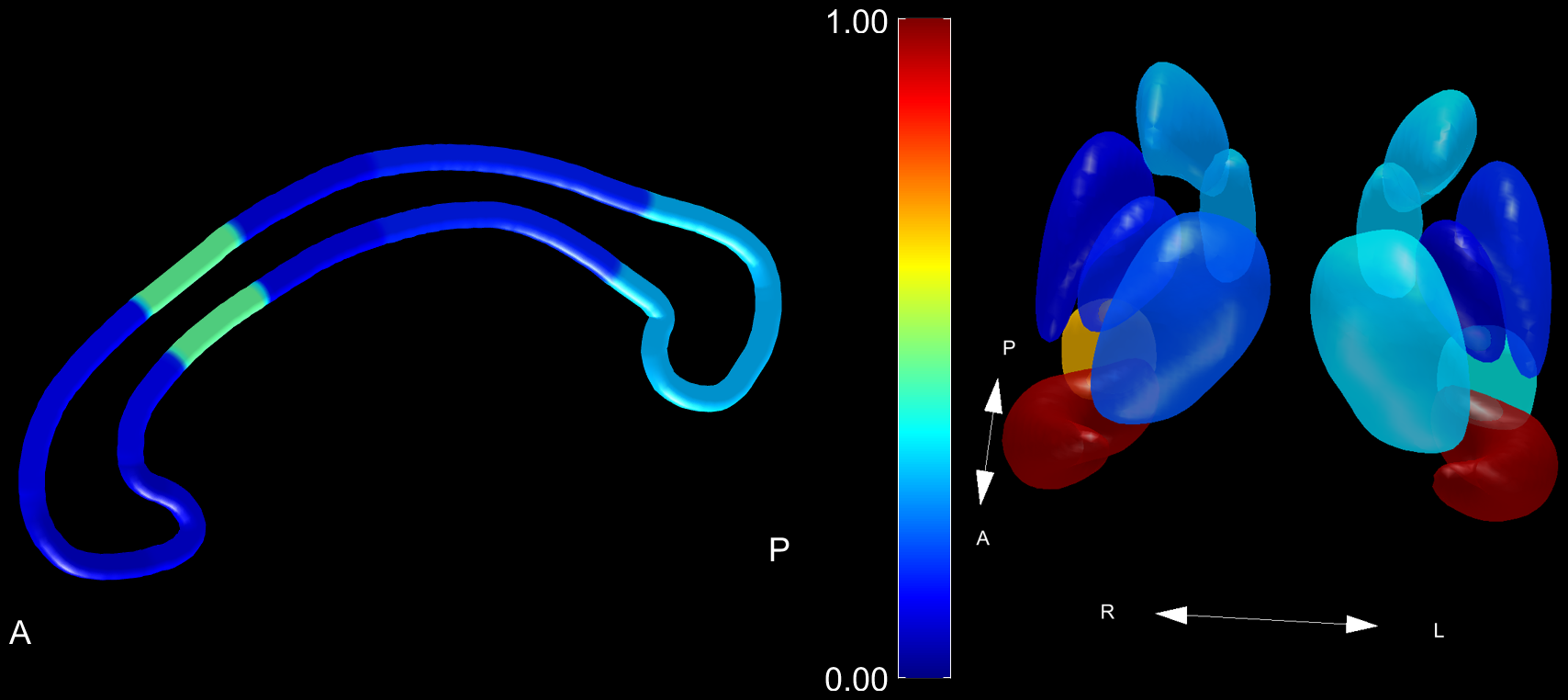


Figure S4: Feature selection probabilities for the CTL/AD cross-validation procedure for the “CCFIRST” feature set. Upper panel: sorted feature selection probabilities, coloured by source feature set. Lower panel: Regional callosal thickness (left) and subcortical grey matter nuclei (right).


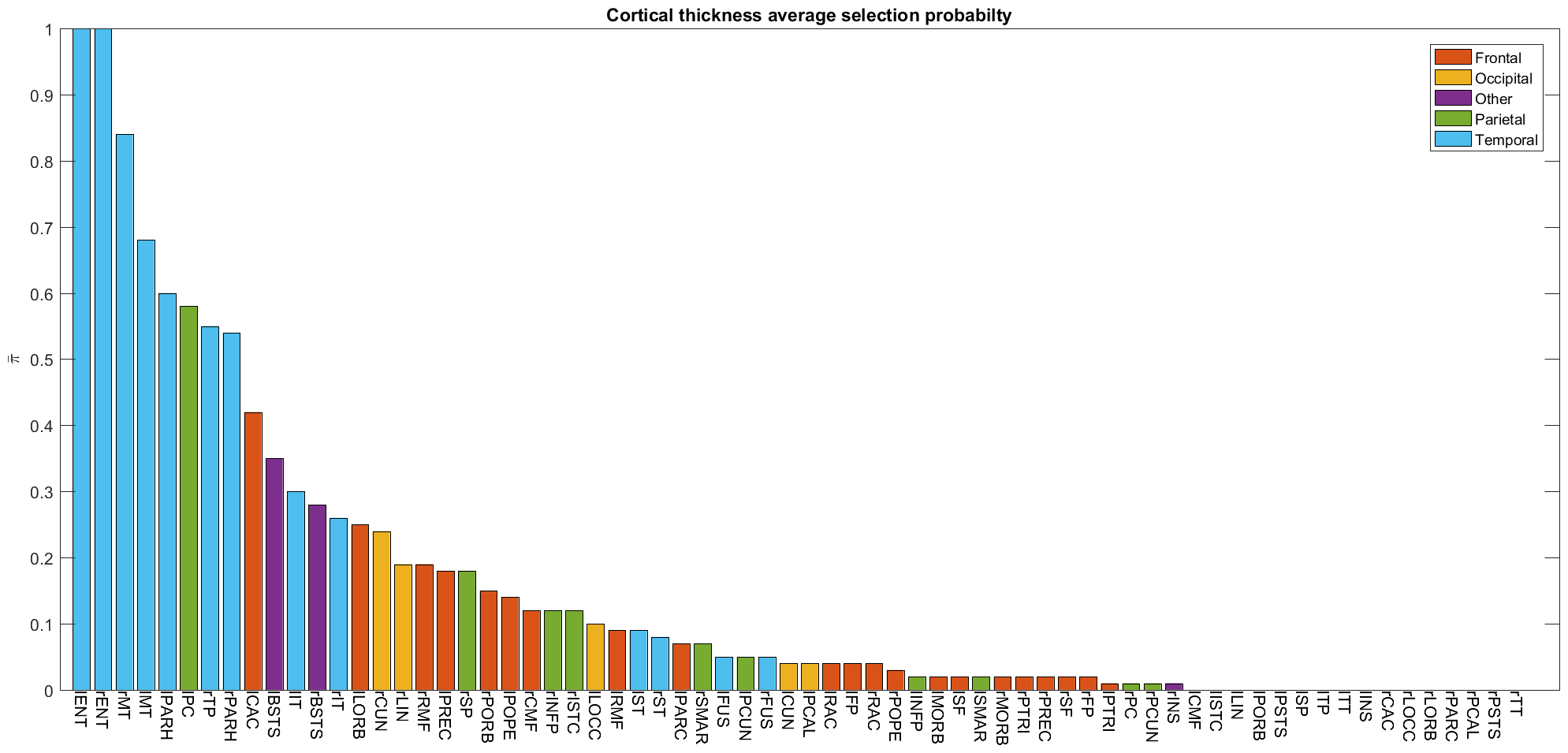

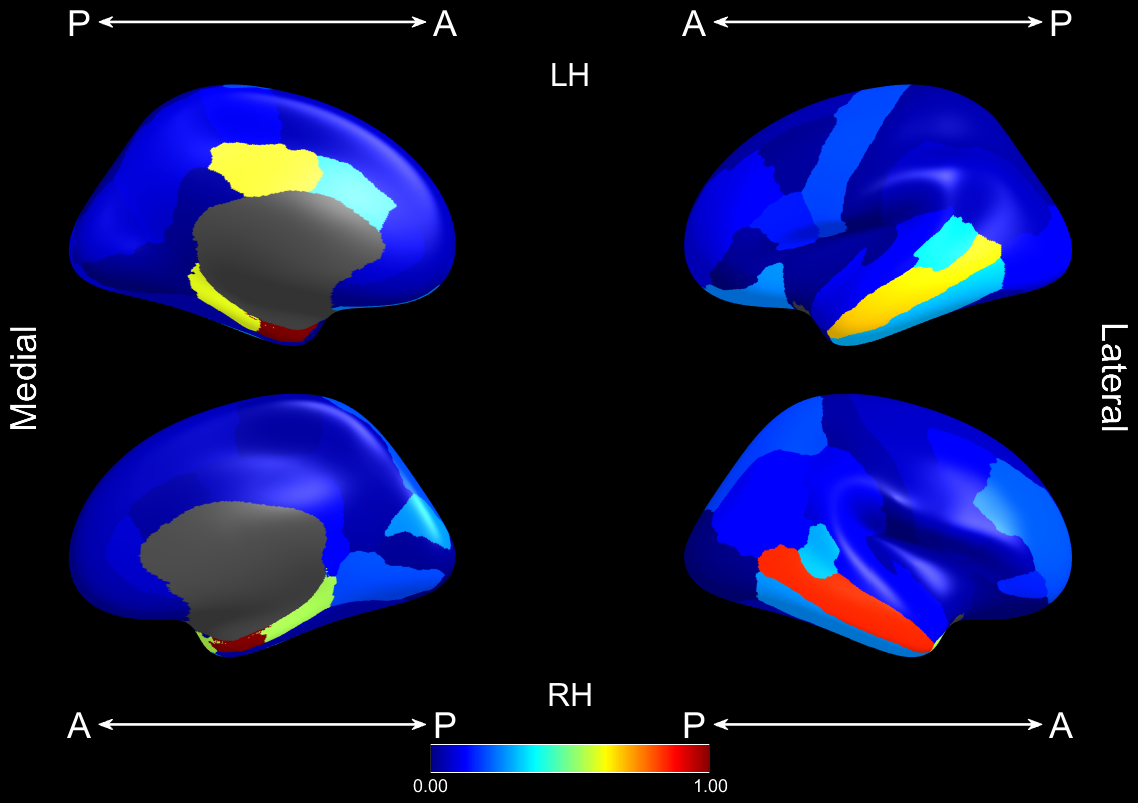


Figure S5: Feature selection probabilities for the CTL/AD cross-validation procedure for the “FS” feature set. Upper panel: sorted feature selection probabilities, coloured by source feature set. Lower panel: cortical thickness.


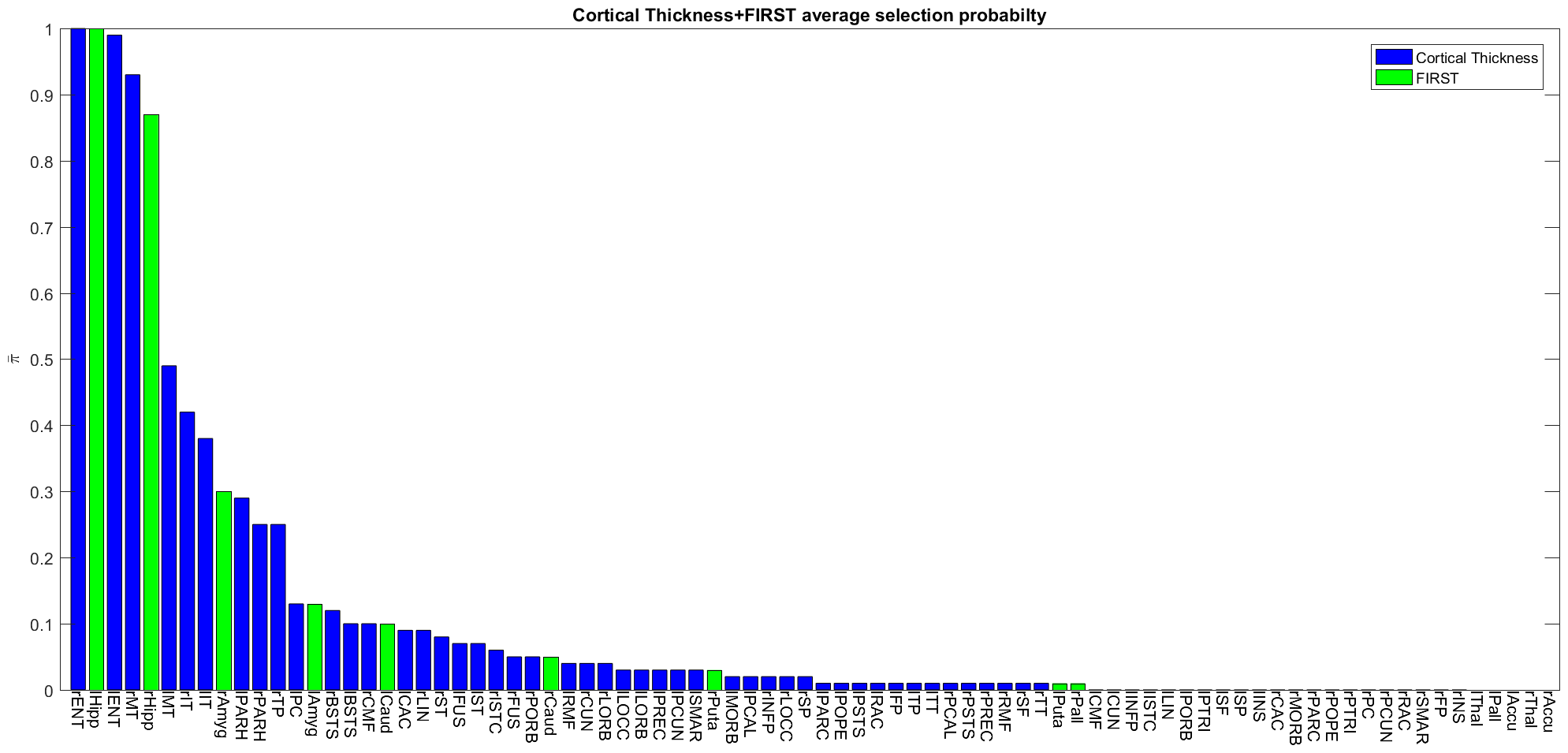

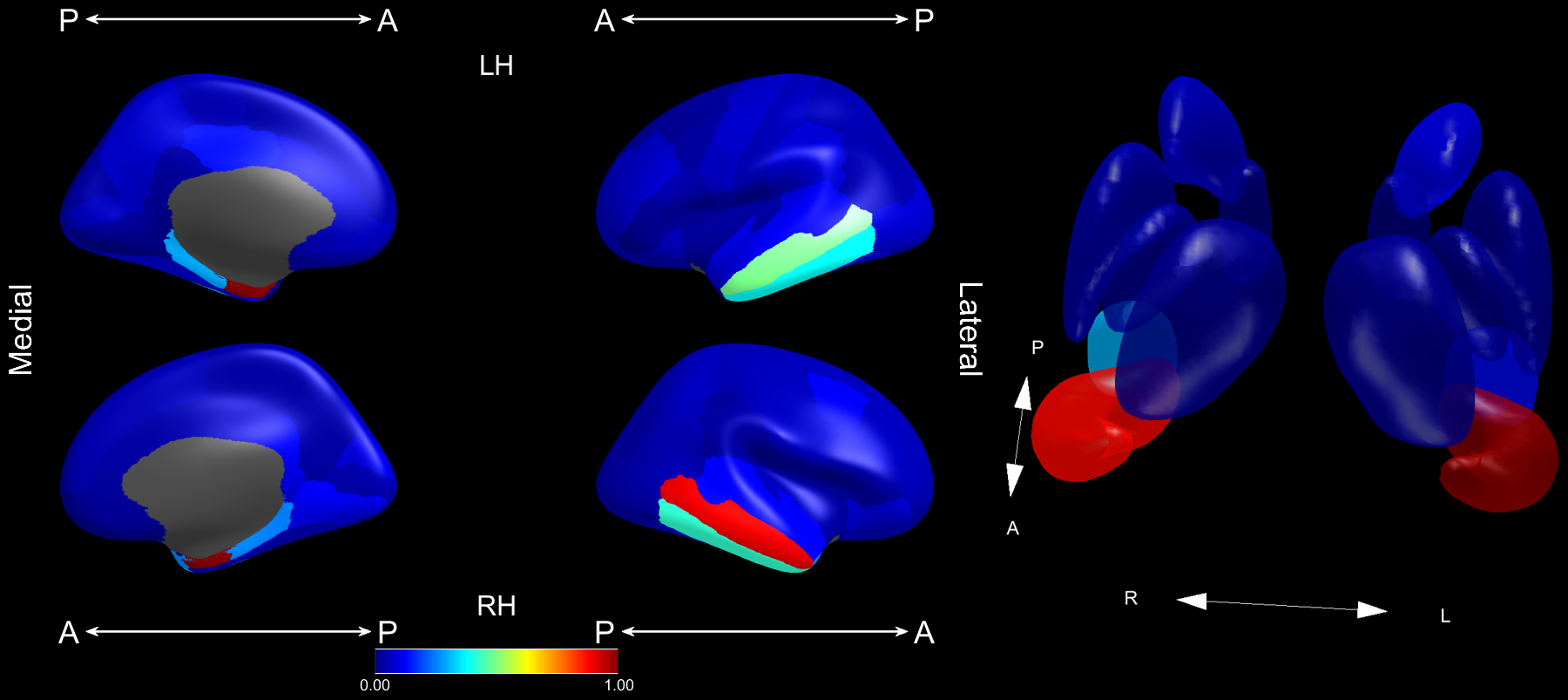


Figure S6: Feature selection probabilities for the CTL/AD cross-validation procedure for the “FSFIRST” feature set. Upper panel: sorted feature selection probabilities, coloured by source feature set. Lower panel: cortical thickness (left) and subcortical grey matter nuclei (right).


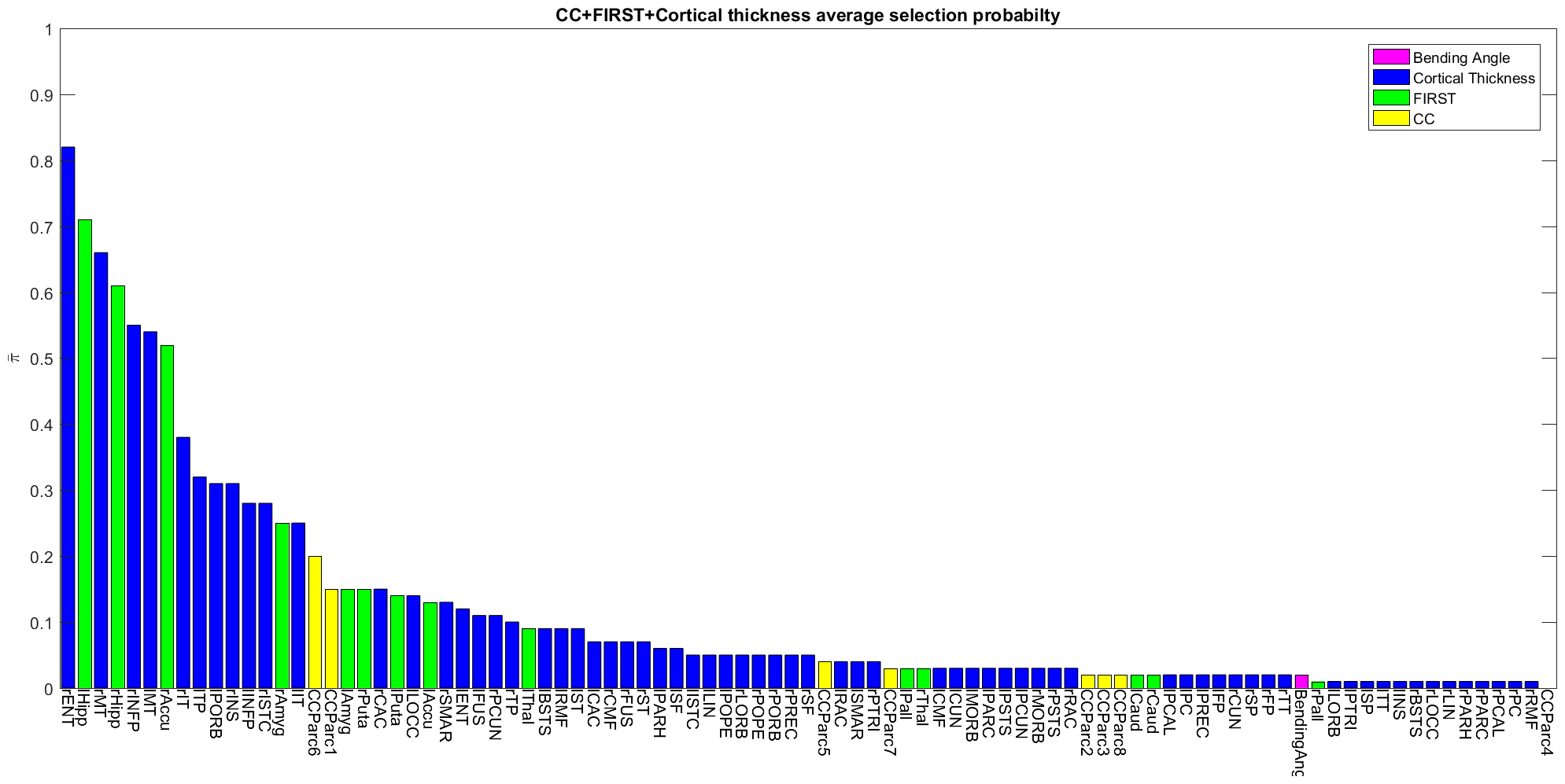

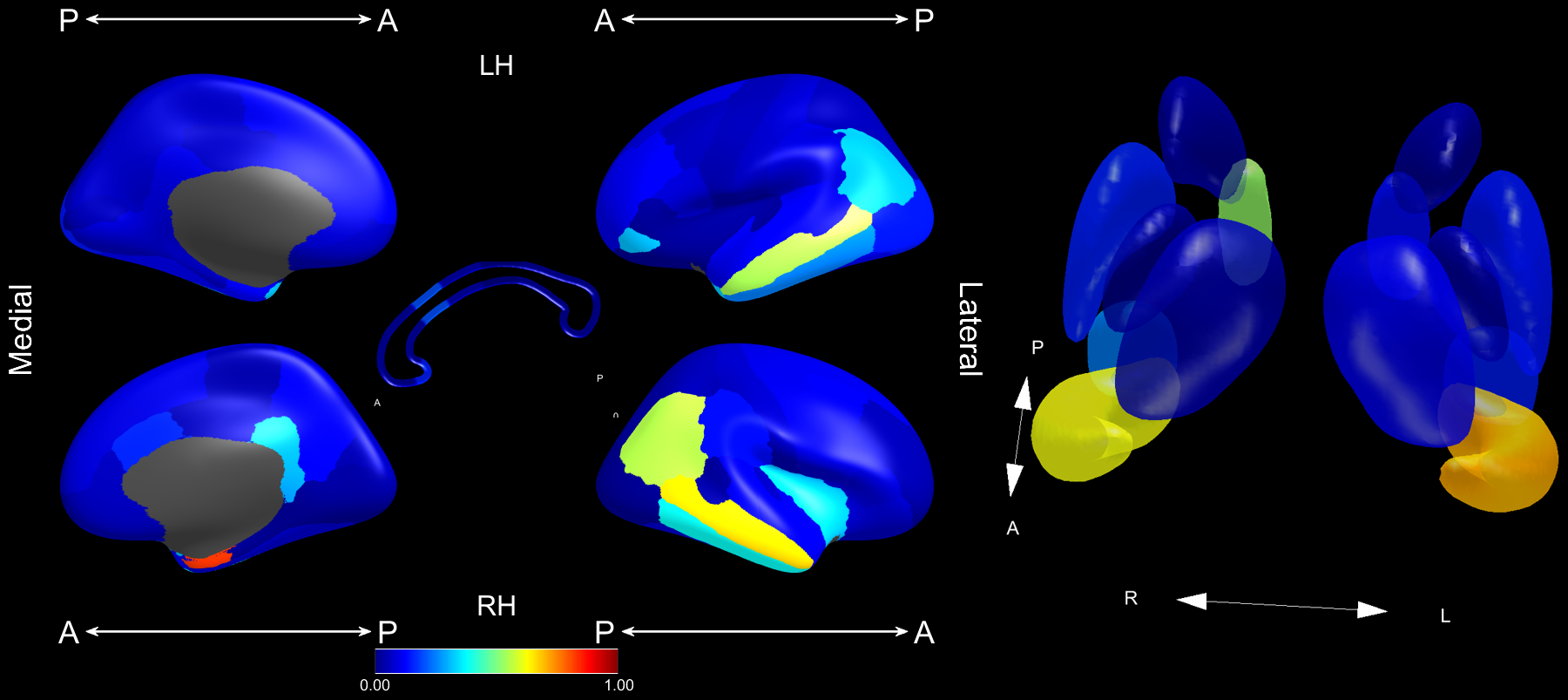


Figure S7: Feature selection probabilities per the cross-validation procedure, by-CDR classification scenario, “ALL” feature set. Upper panel: sorted feature selection probabilities, coloured by source feature set. Lower panel: cortical thickness and regional callosal thickness (left) and subcortical grey matter nuclei (right).


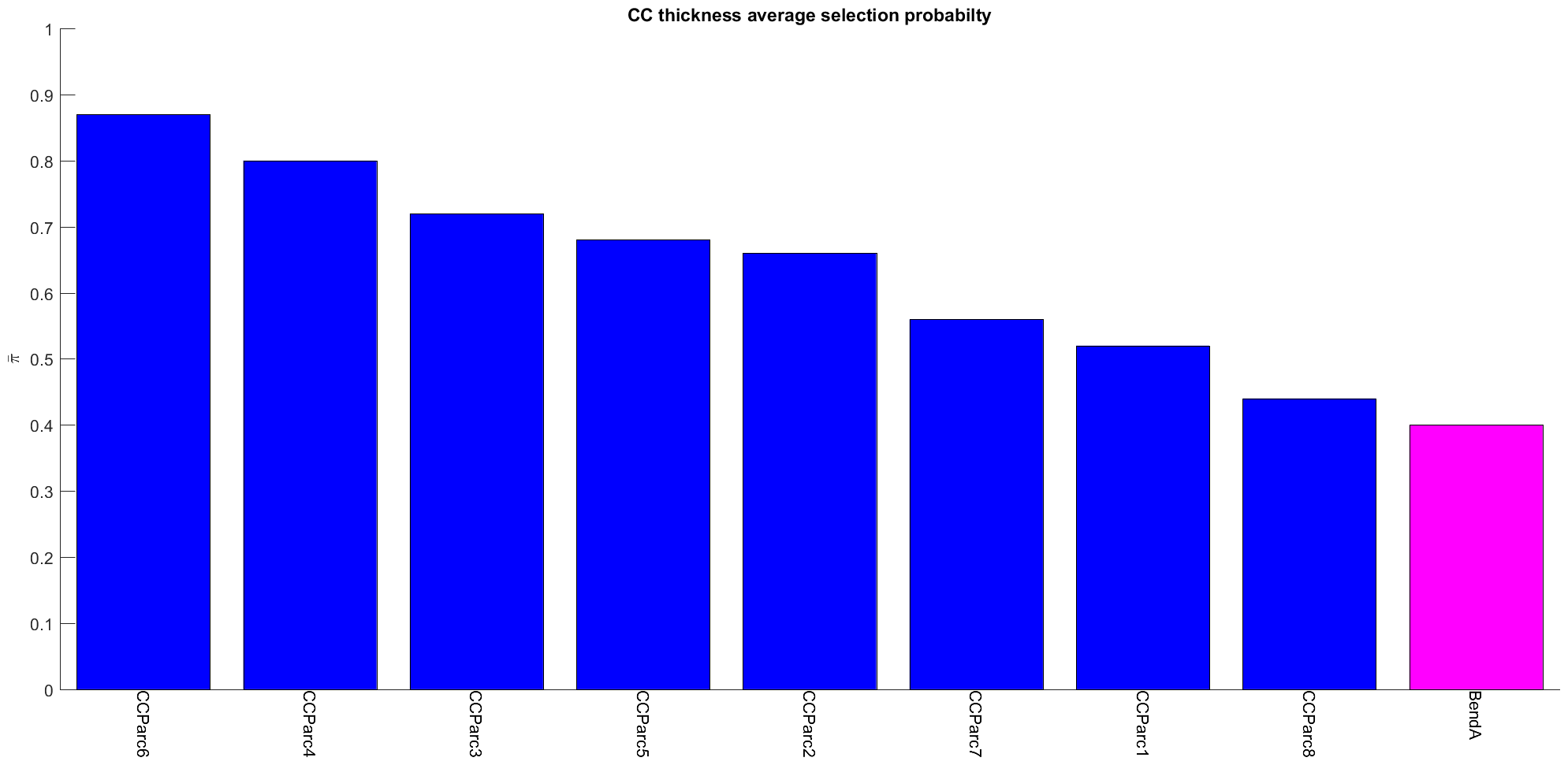

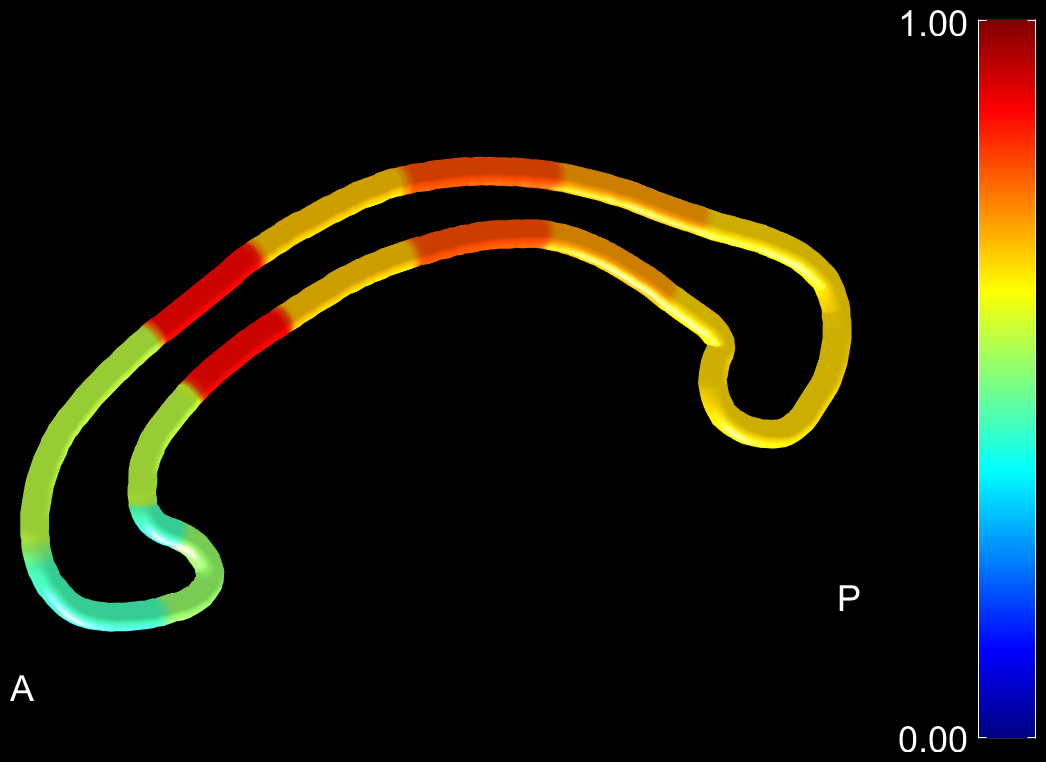


Figure S8: Feature selection probabilities per the cross-validation procedure, by-CDR classification scenario, “CC” feature set. Upper panel: sorted feature selection probabilities. Lower panel: Regional callosal thickness.


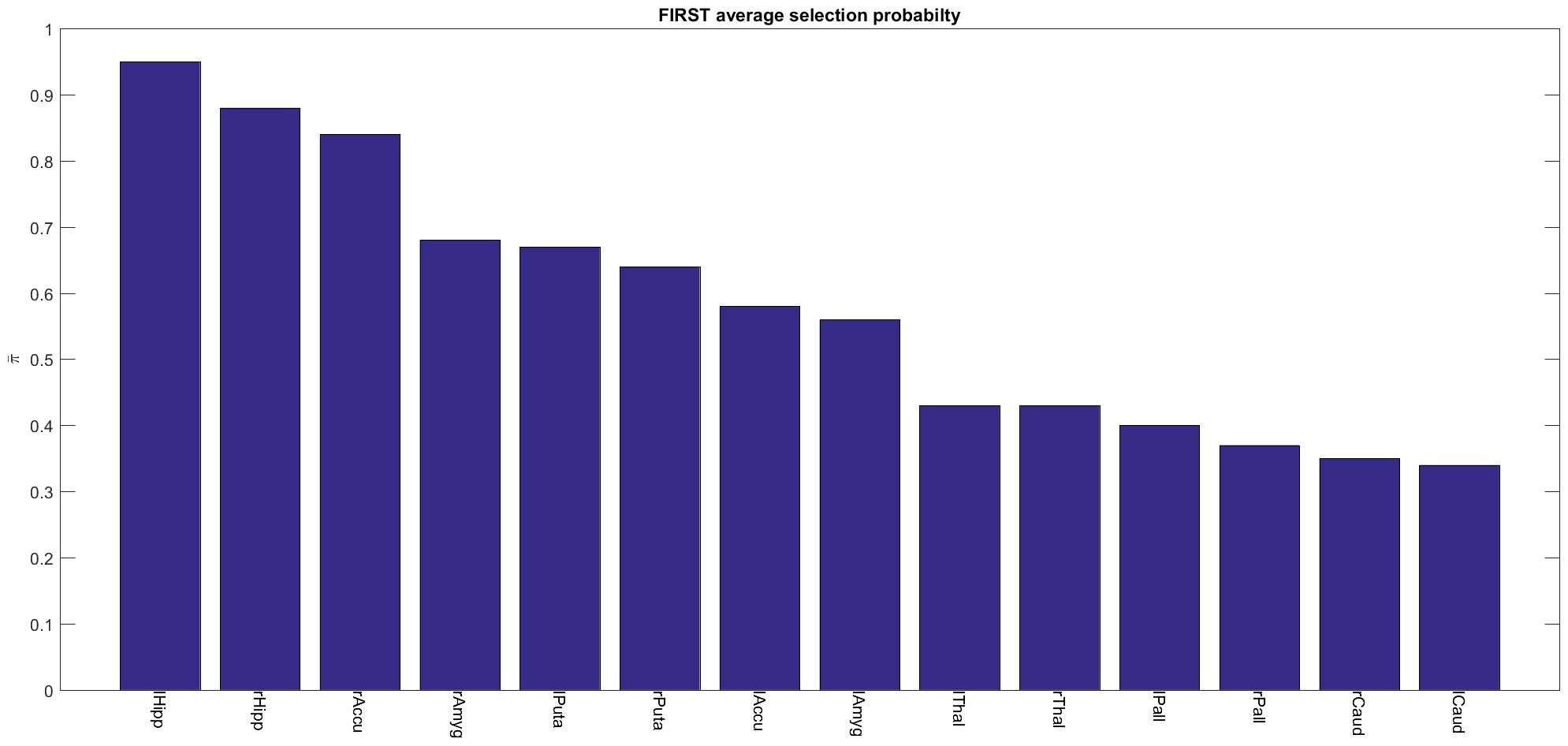

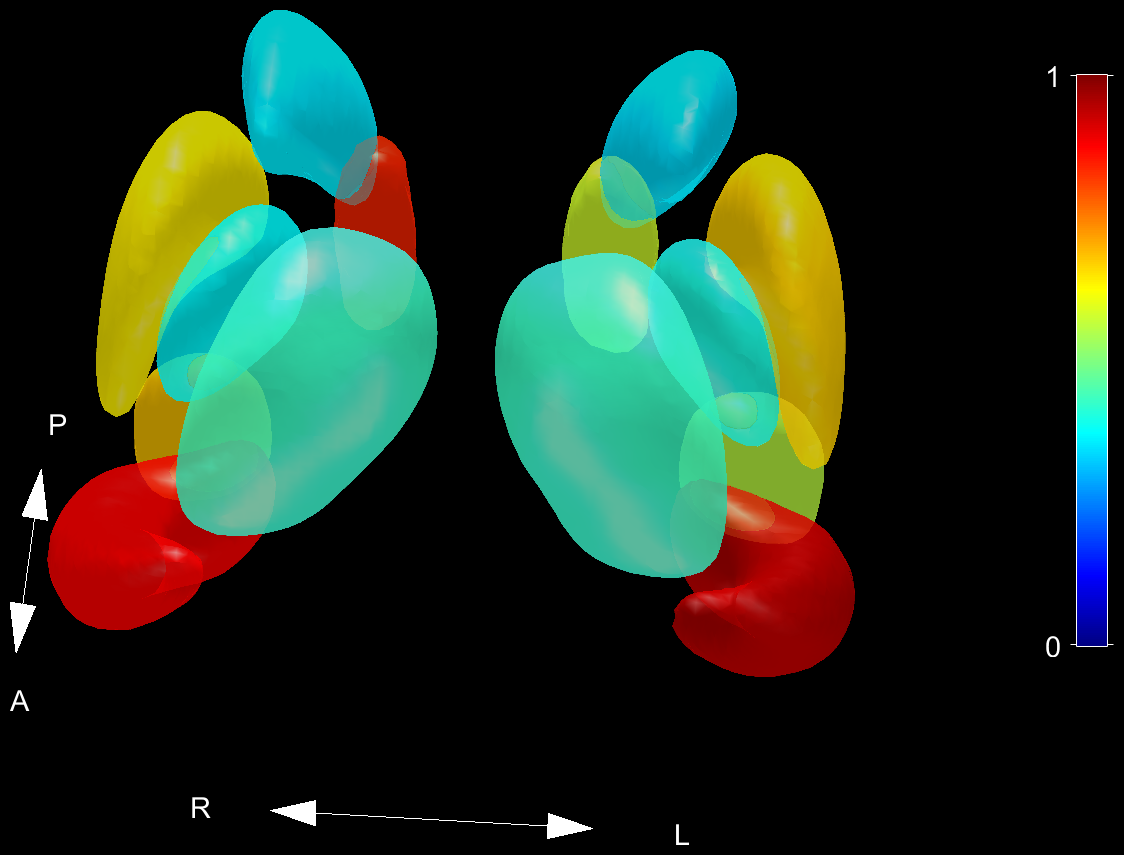


Figure S9: Feature selection probabilities per the cross-validation procedure, by-CDR classification scenario, “FIRST” feature set. Upper panel: sorted feature selection probabilities, coloured by source feature set. Lower panel: subcortical grey matter nuclei.


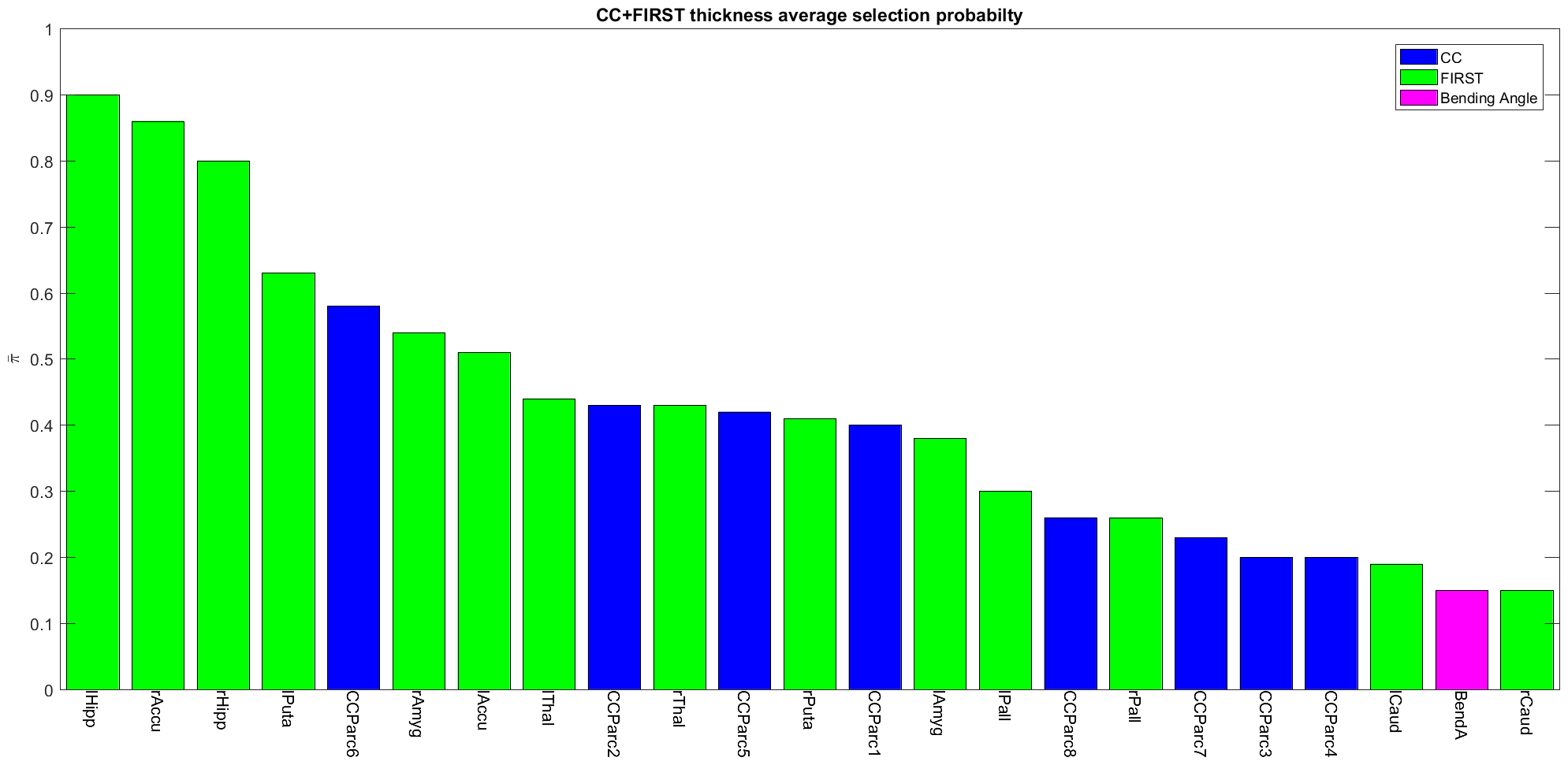

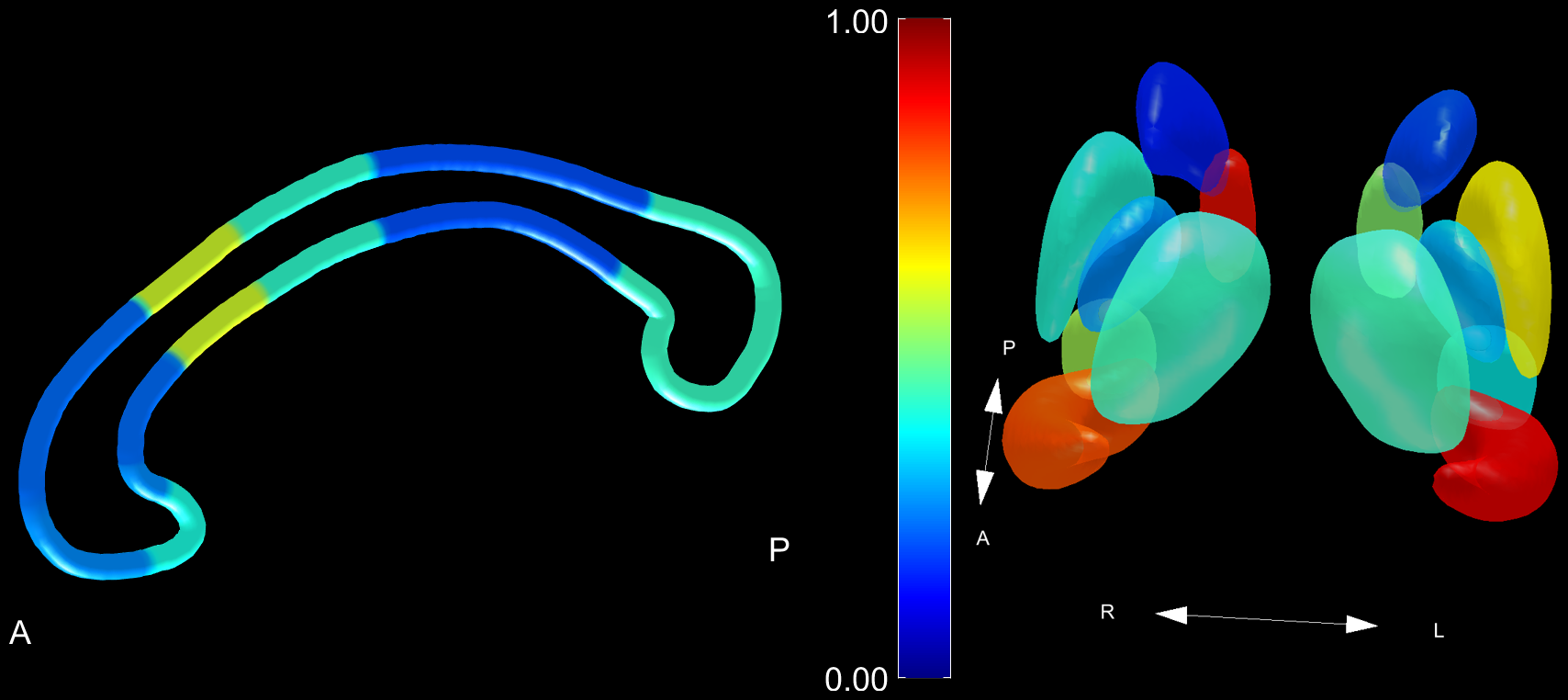


Figure S10: Feature selection probabilities per the cross-validation procedure, by-CDR classification scenario, “CCFIRST” feature set. Upper panel: sorted feature selection probabilities, coloured by source feature set. Lower panel: Regional callosal thickness (left) and subcortical grey matter nuclei (right).


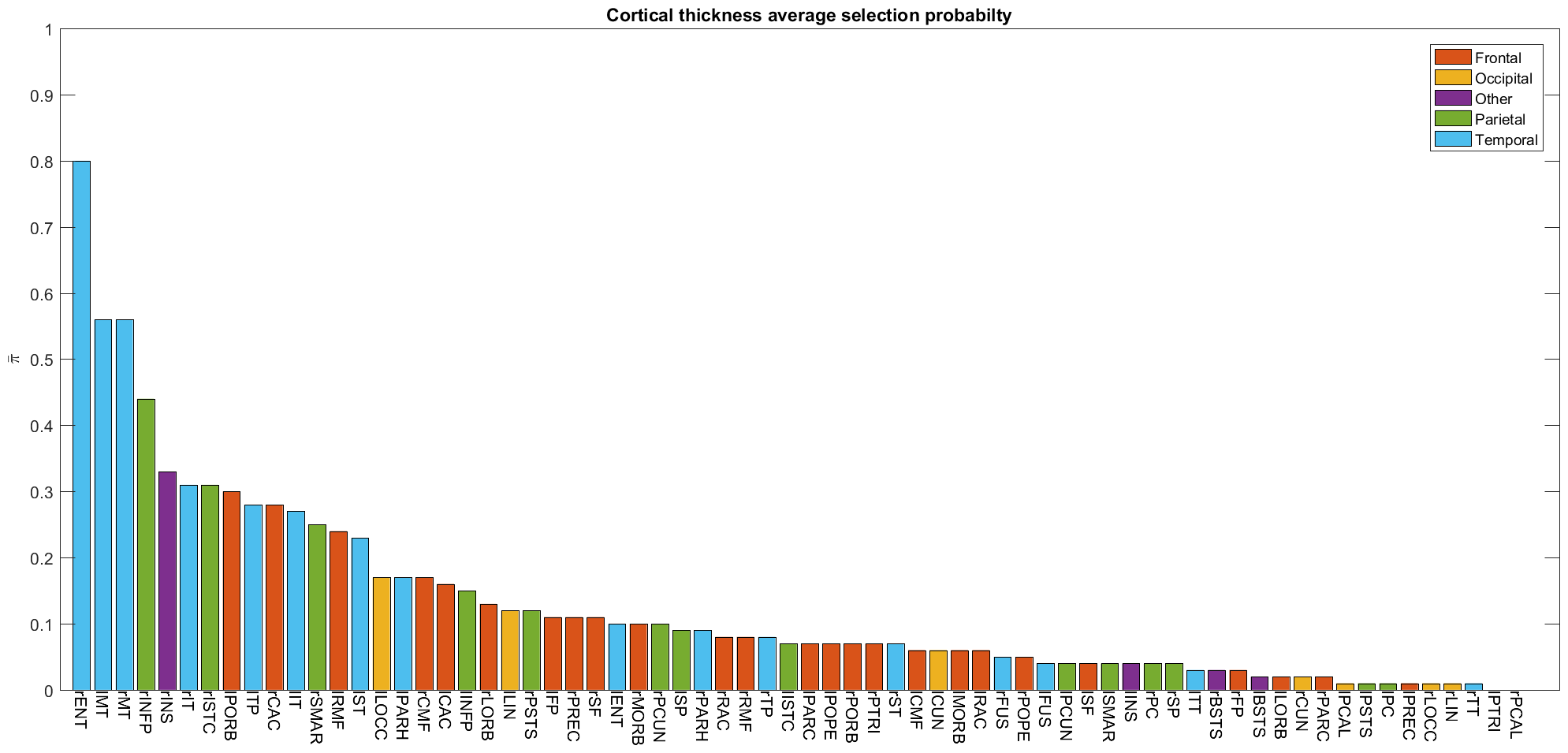

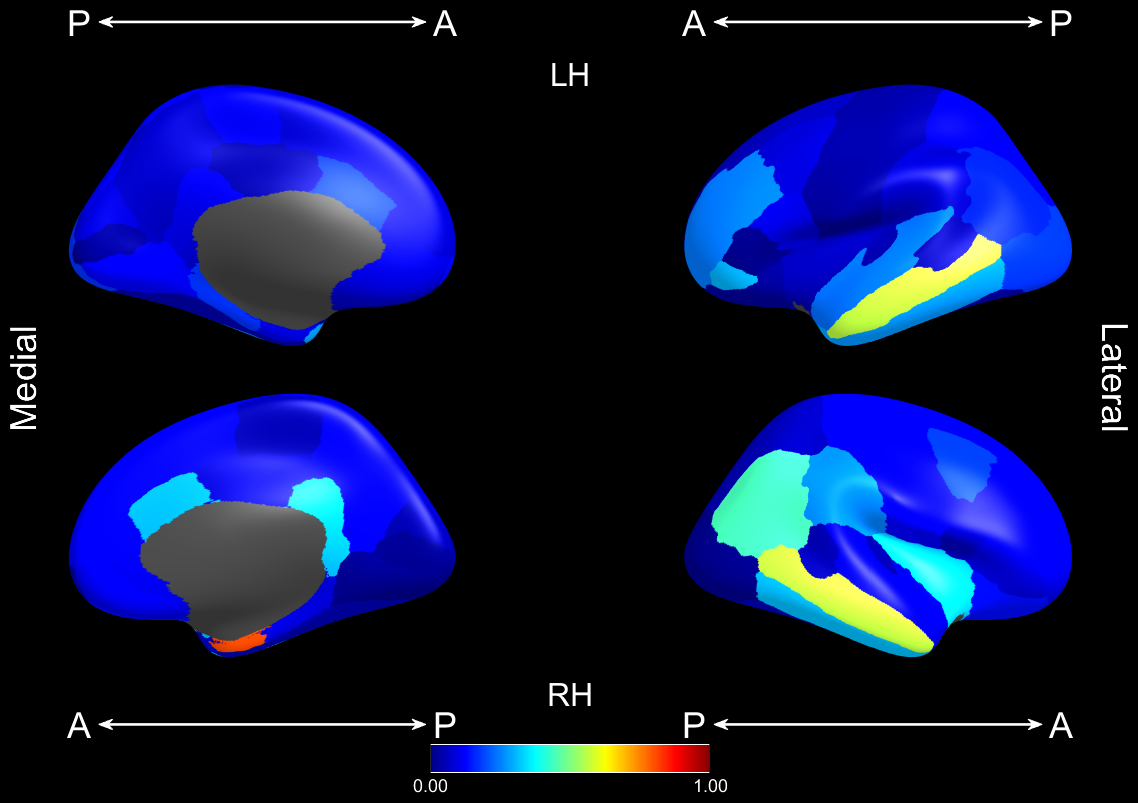


Figure S11: Feature selection probabilities per the cross-validation procedure, by-CDR classification scenario, “FS” feature set. Upper panel: sorted feature selection probabilities, coloured by source feature set. Lower panel: cortical thickness.


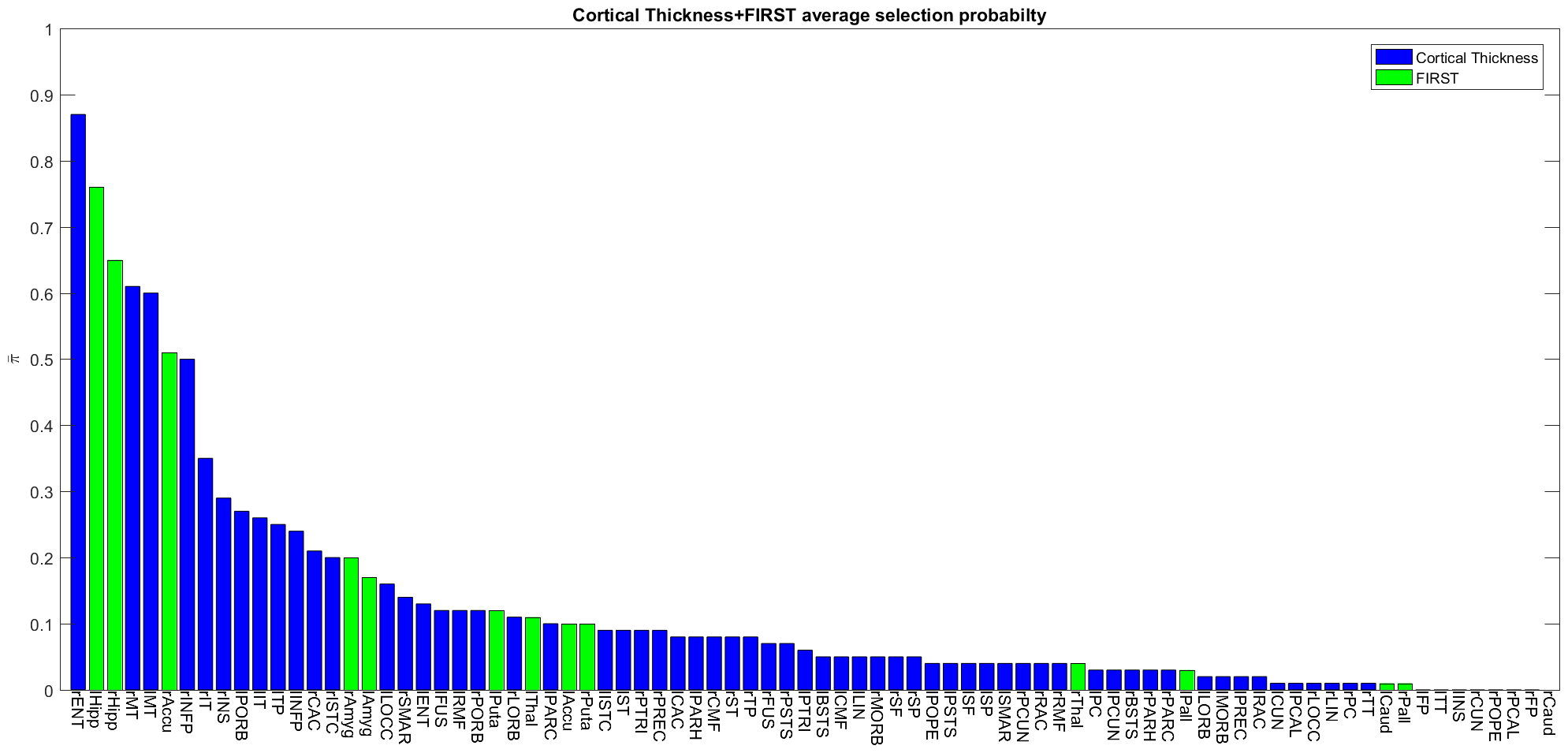

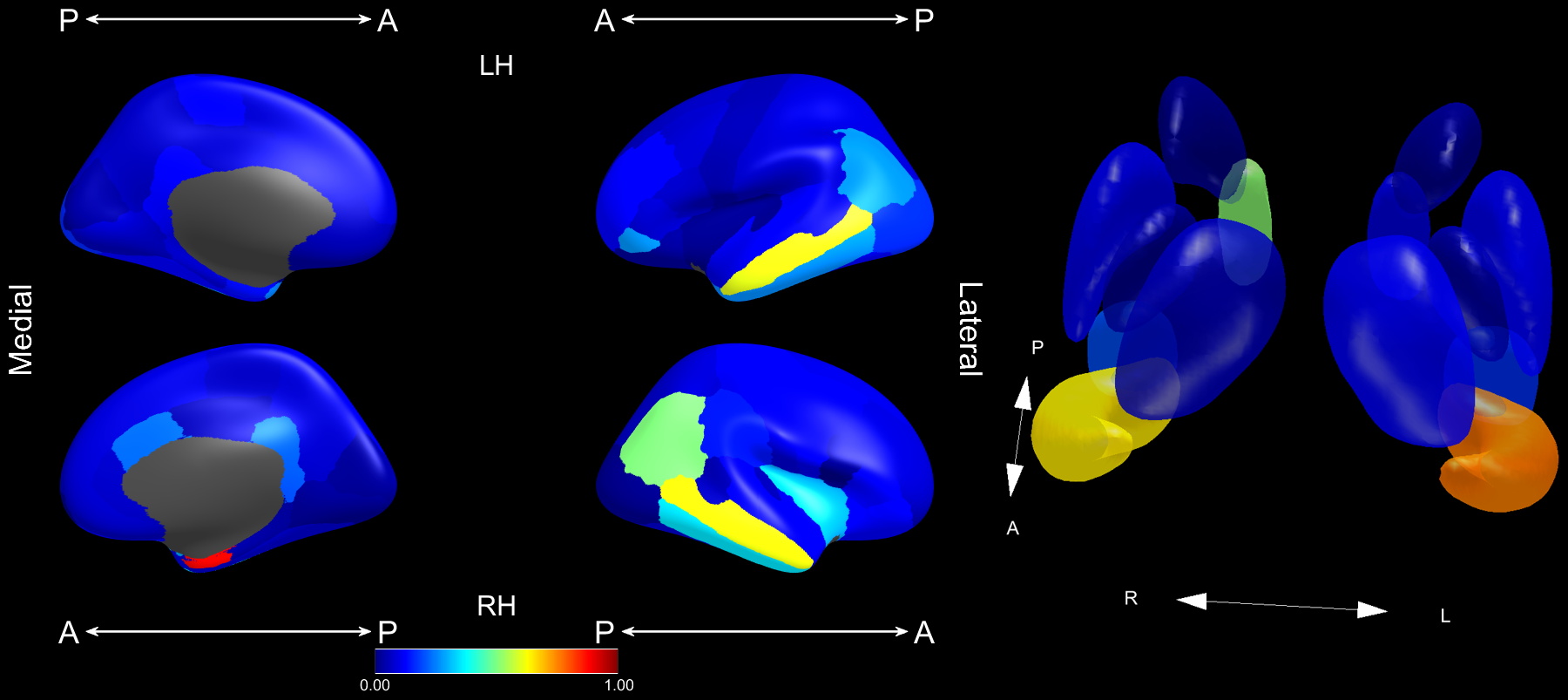


Figure S12: Feature selection probabilities per the cross-validation procedure, by-CDR classification scenario, “FSFIRST” feature set. Upper panel: sorted feature selection probabilities, coloured by source feature set. Lower panel: cortical thickness (left) and subcortical grey matter nuclei (right).


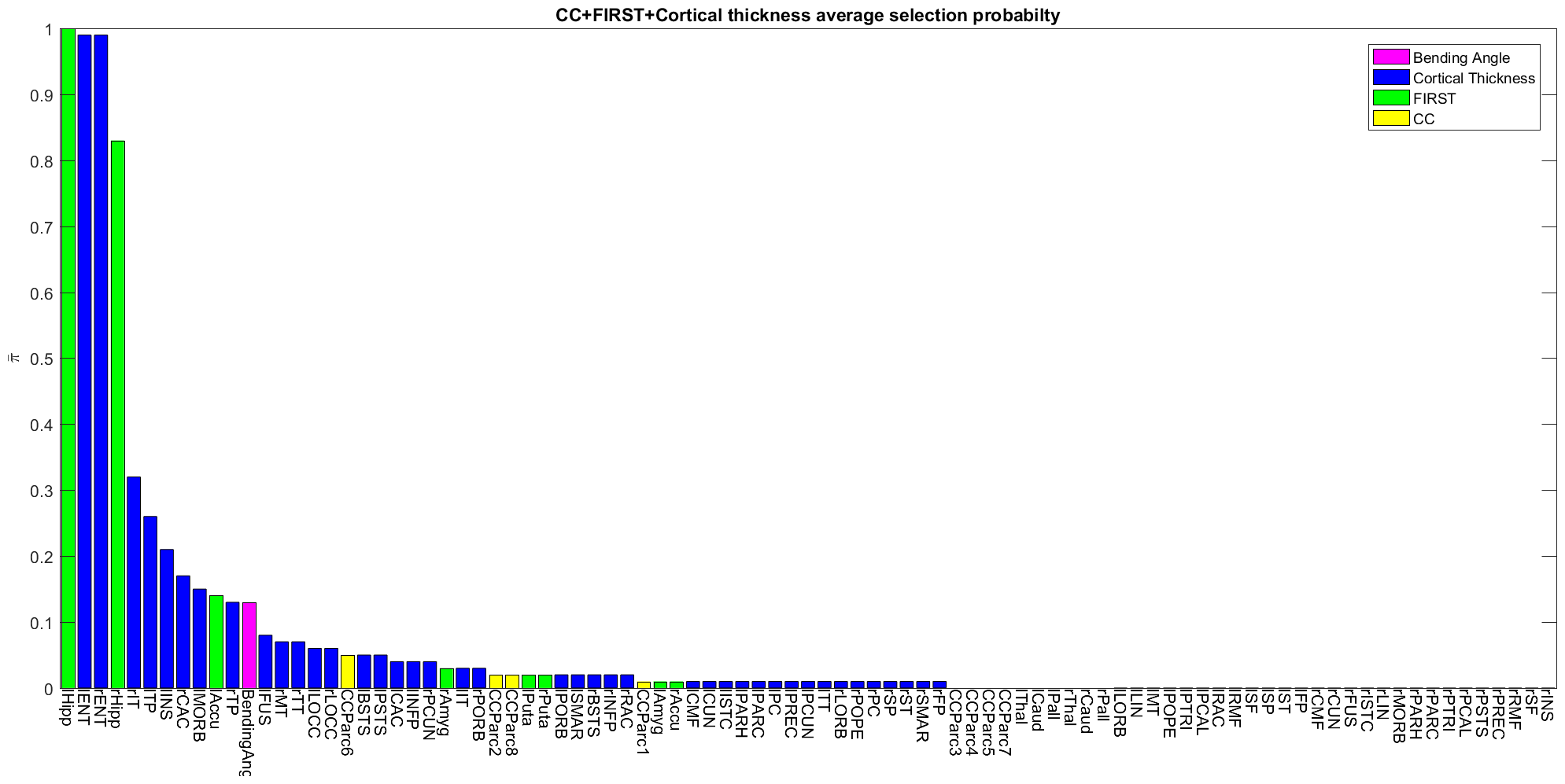

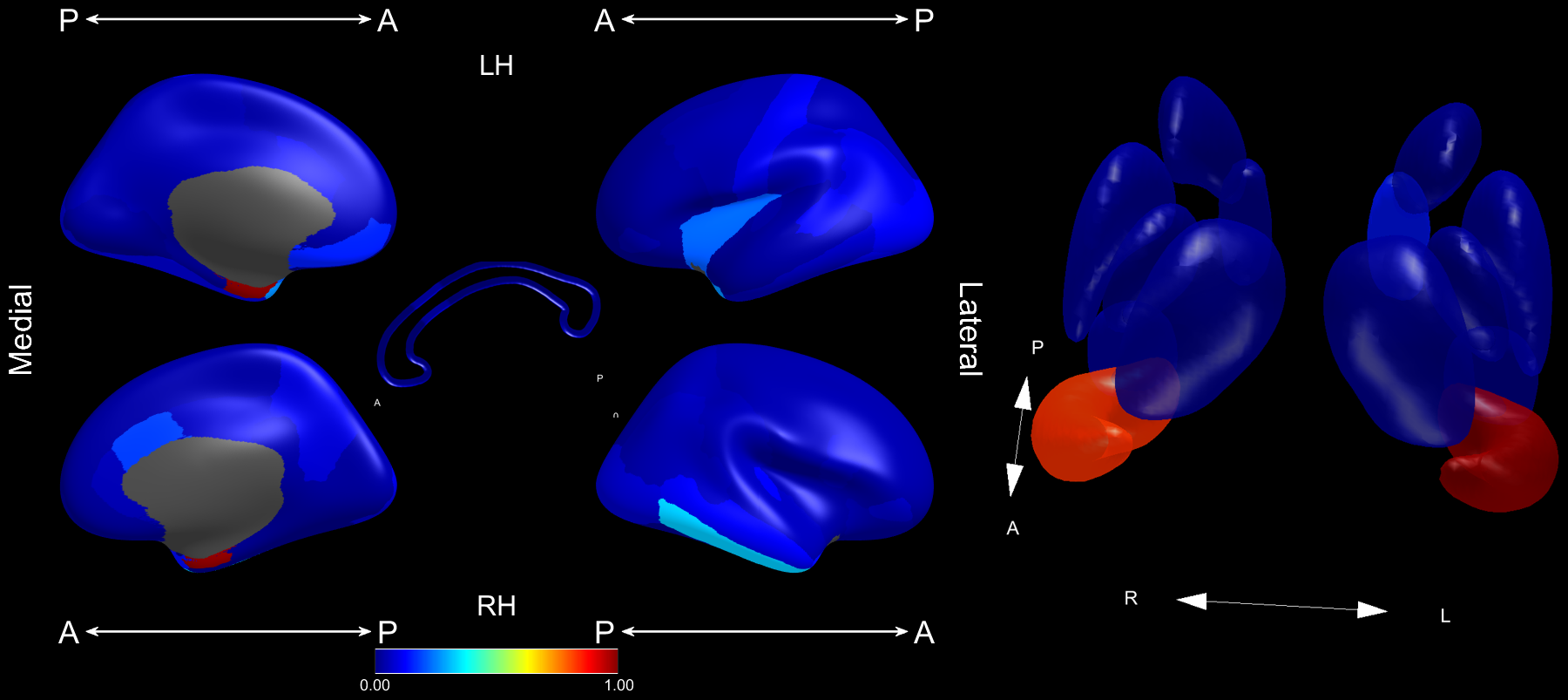


Figure S13: Feature selection probabilities per the cross-validation procedure, by-Trajectory classification scenario, “ALL” feature set. Upper panel: sorted feature selection probabilities, coloured by source feature set. Lower panel: cortical thickness and regional callosal thickness (left) and subcortical grey matter nuclei (right).


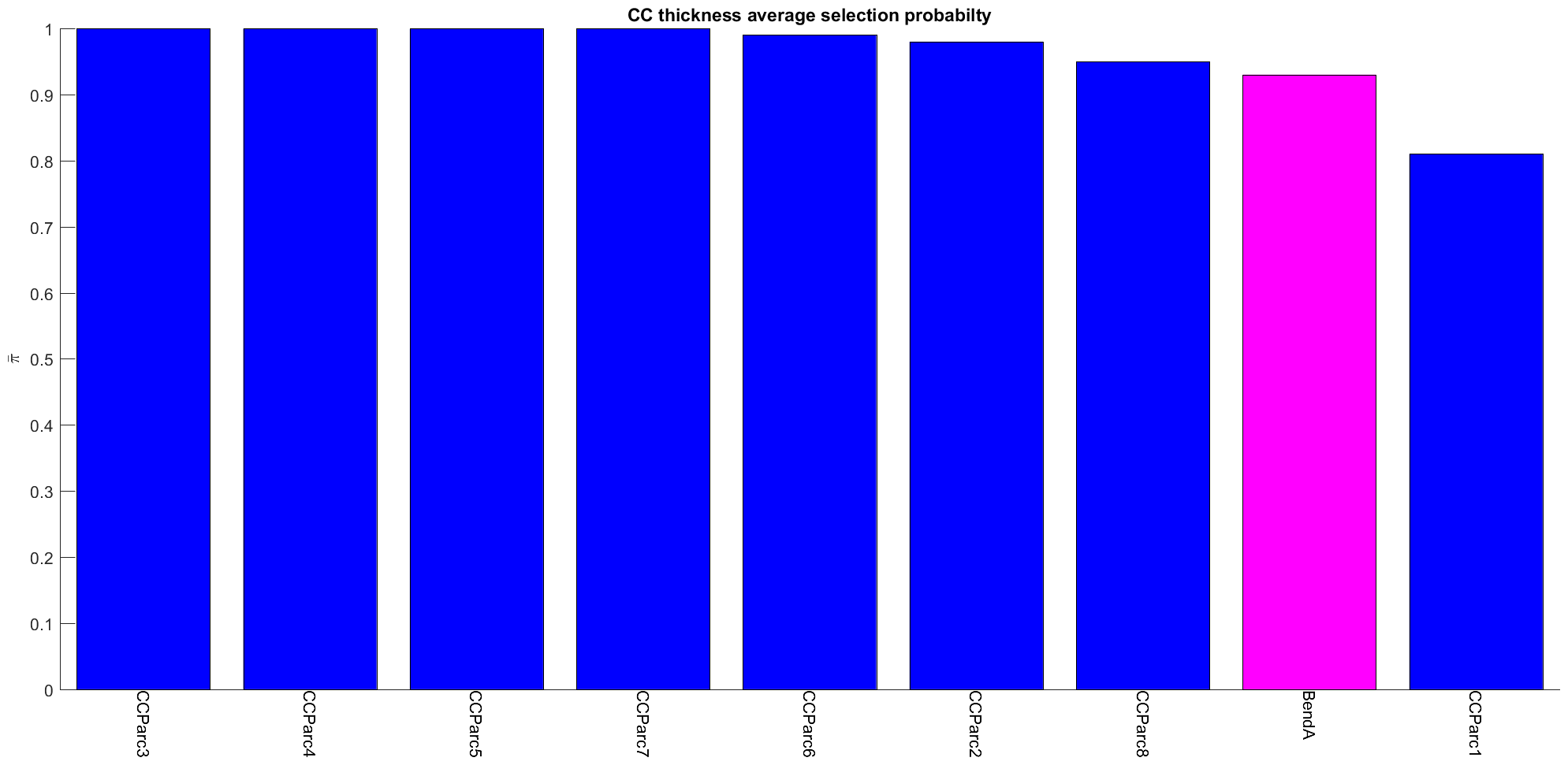

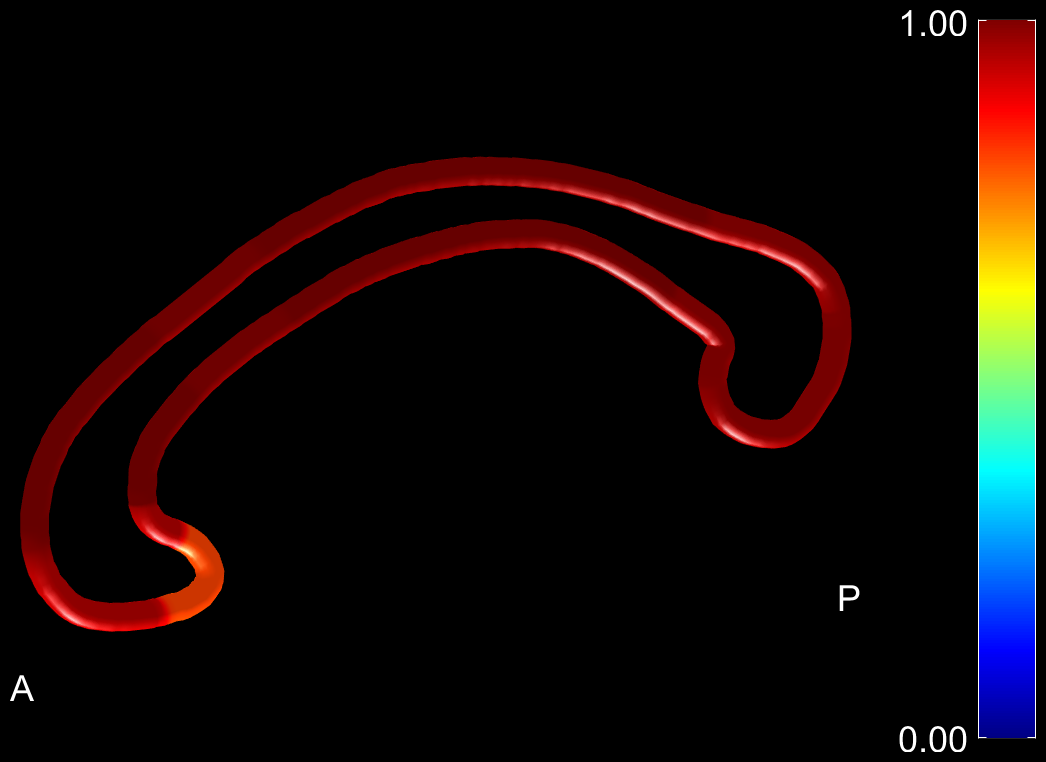


Figure S14: Feature selection probabilities per the cross-validation procedure, by-Trajectory classification scenario, “CC” feature set. Upper panel: sorted feature selection probabilities. Lower panel: Regional callosal thickness.


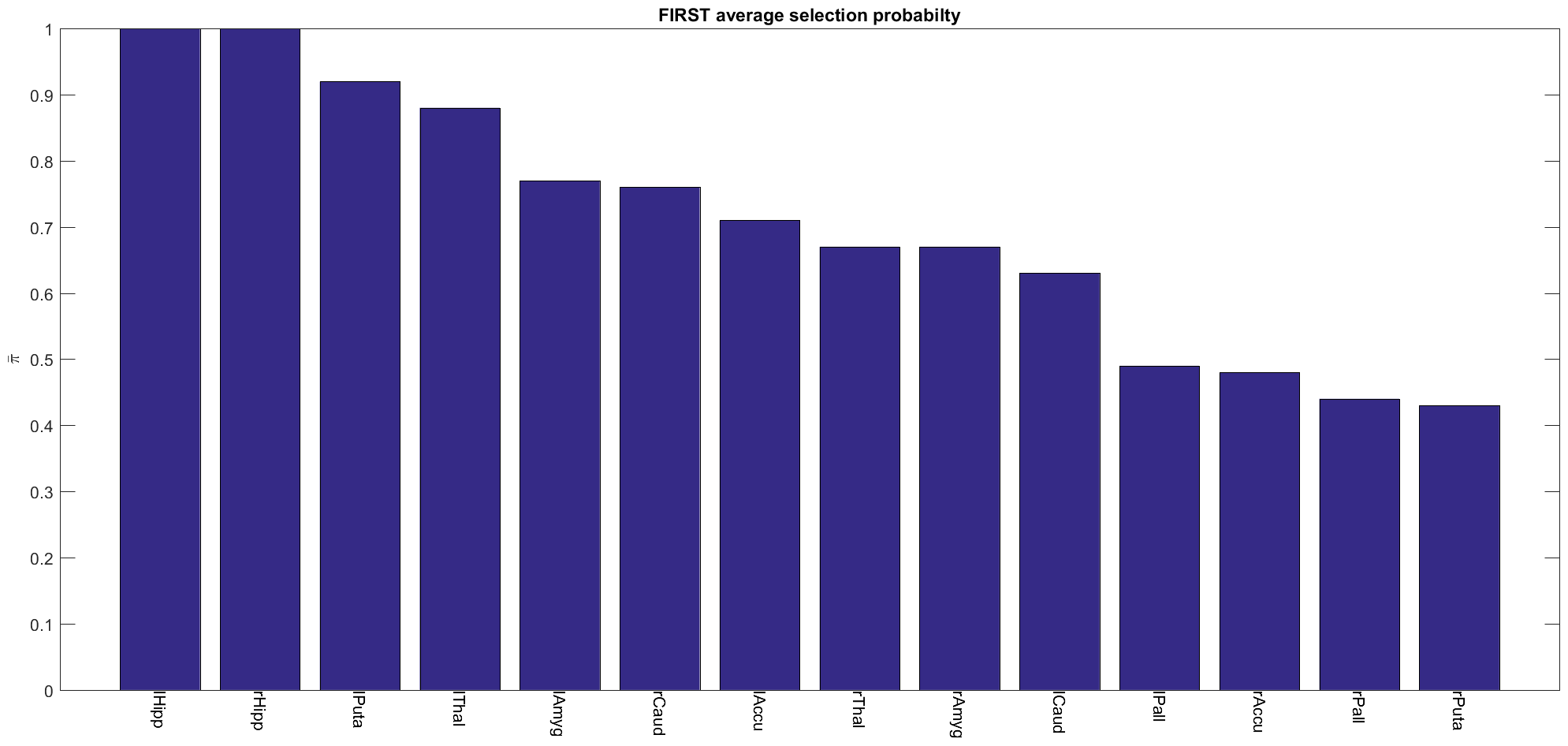

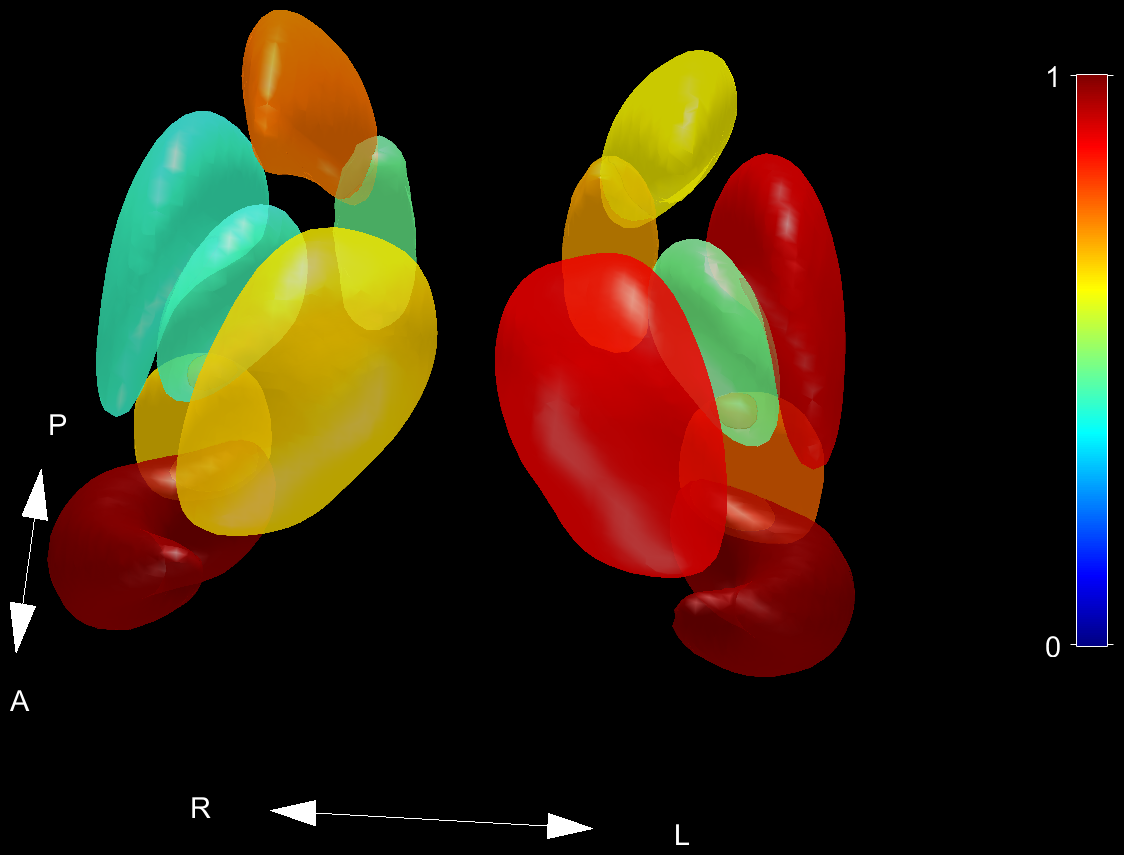


Figure S15: Feature selection probabilities per the cross-validation procedure, by-Trajectory classification scenario, “FIRST” feature set. Upper panel: sorted feature selection probabilities, coloured by source feature set. Lower panel: subcortical grey matter nuclei.


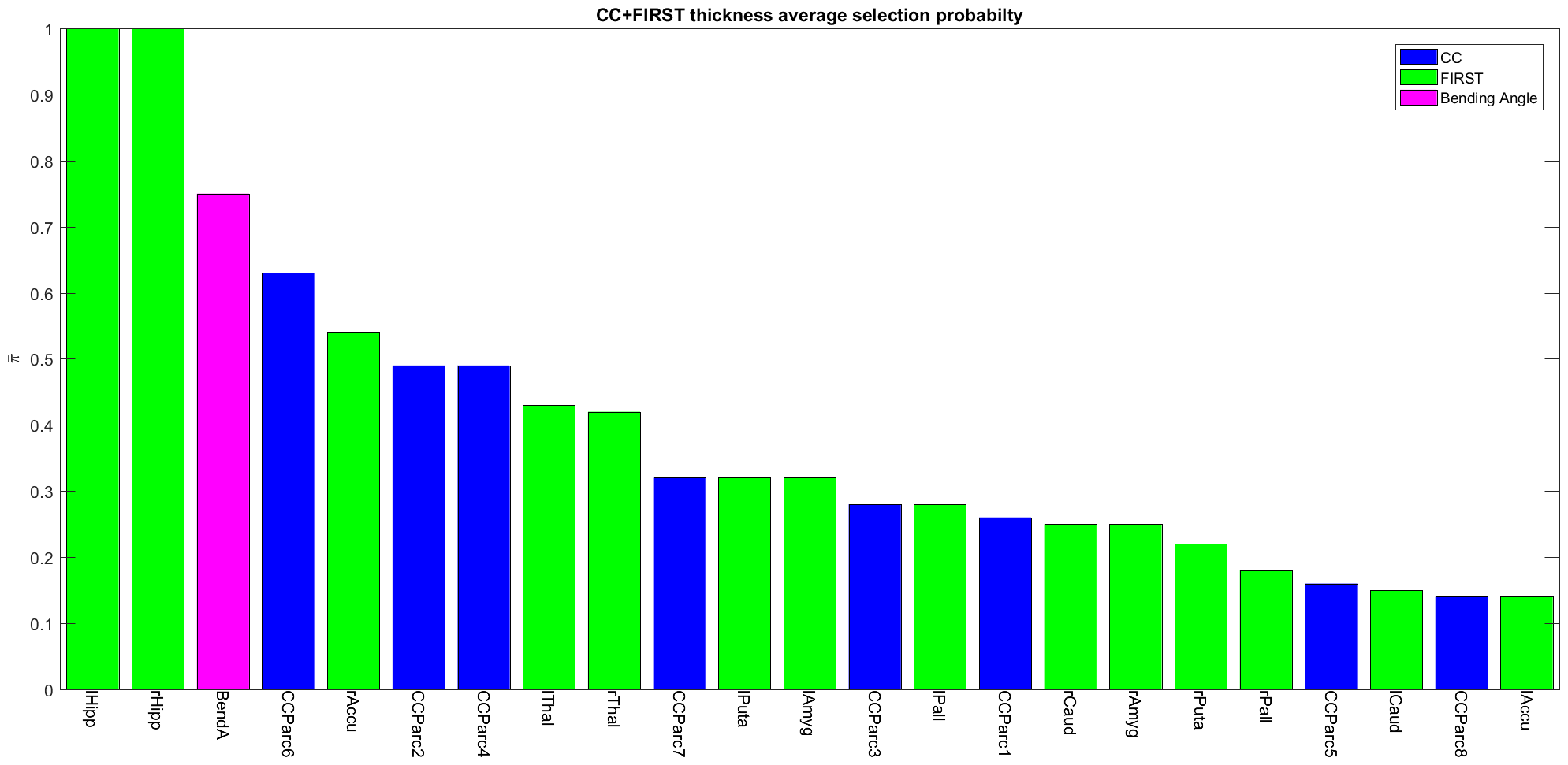

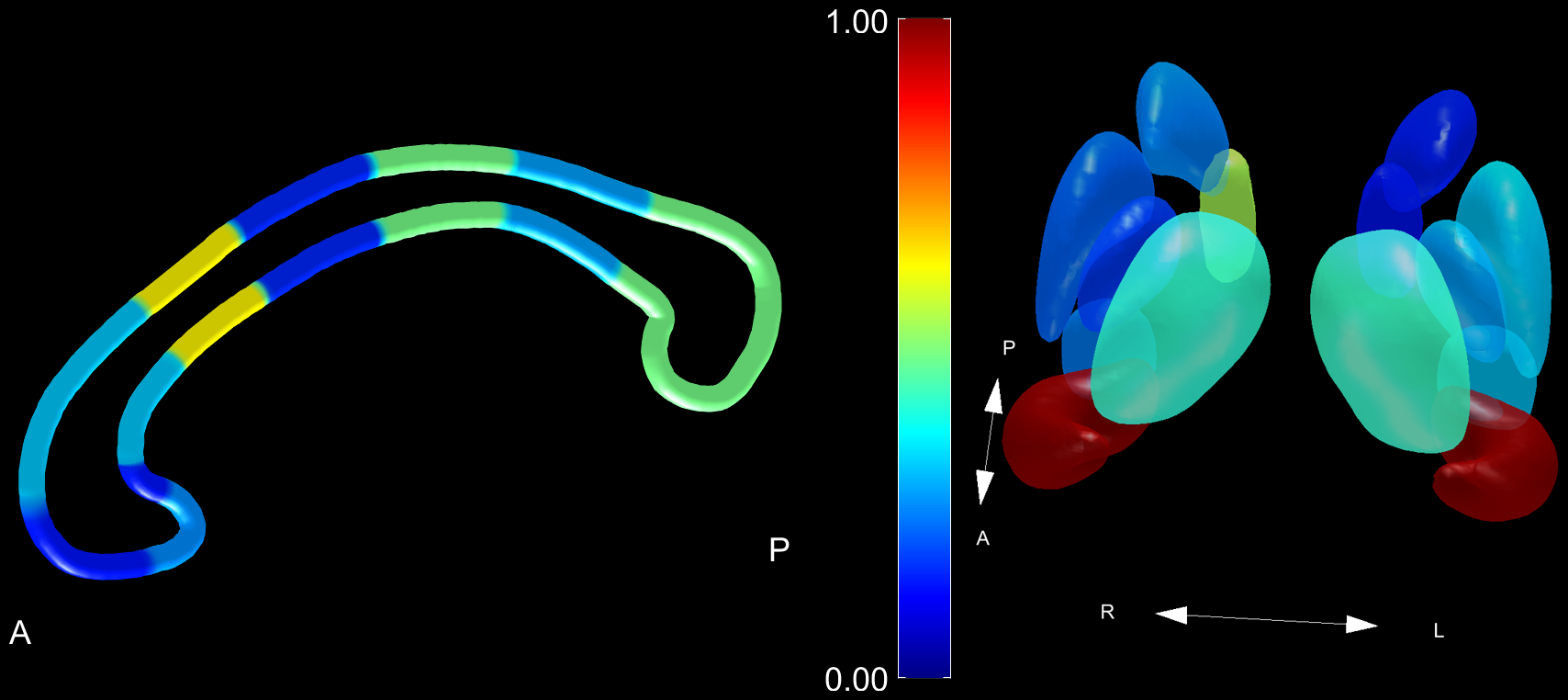


Figure S16: Feature selection probabilities per the cross-validation procedure, by-Trajectory classification scenario, “CCFIRST” feature set. Upper panel: sorted feature selection probabilities, coloured by source feature set. Lower panel: Regional callosal thickness (left) and subcortical grey matter nuclei (right).


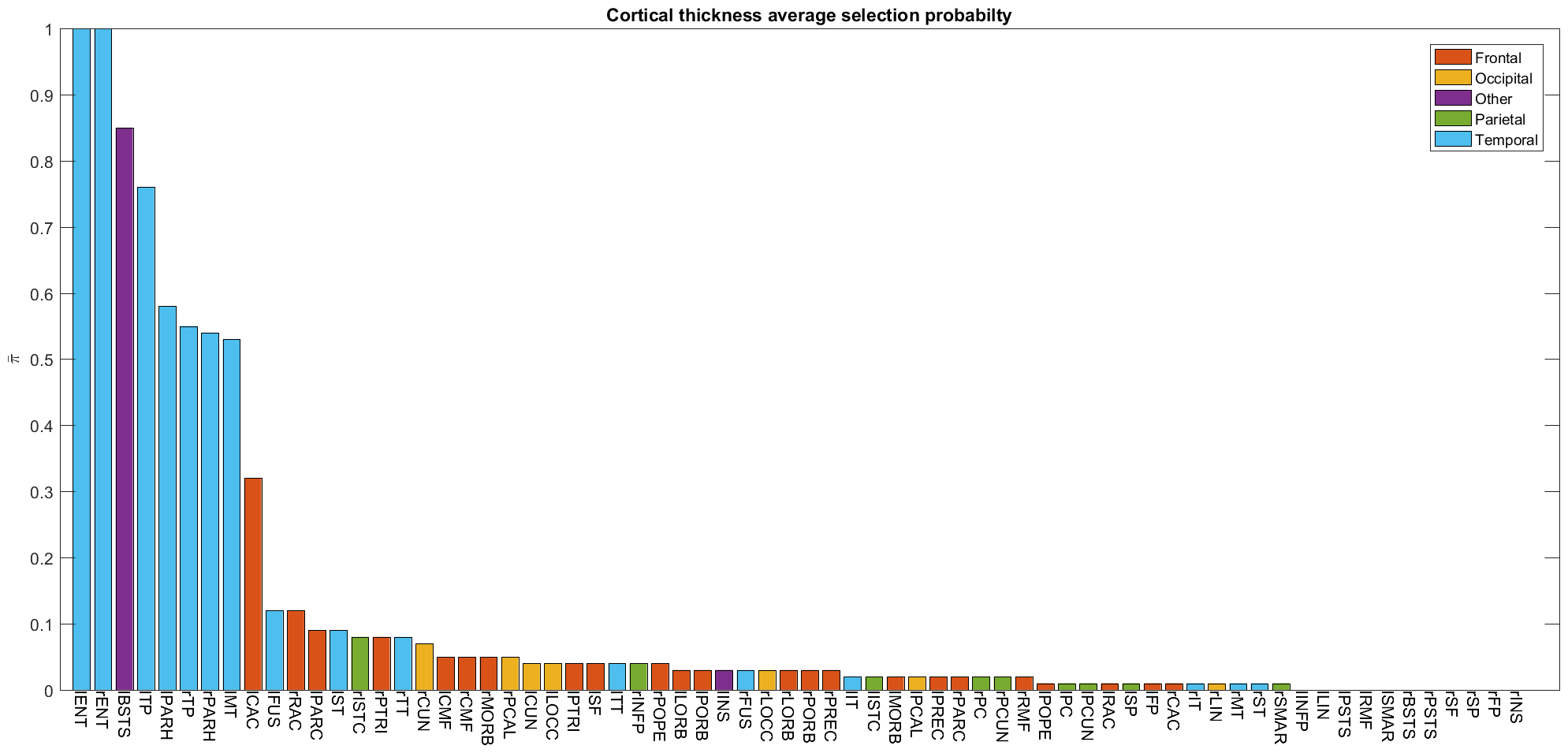

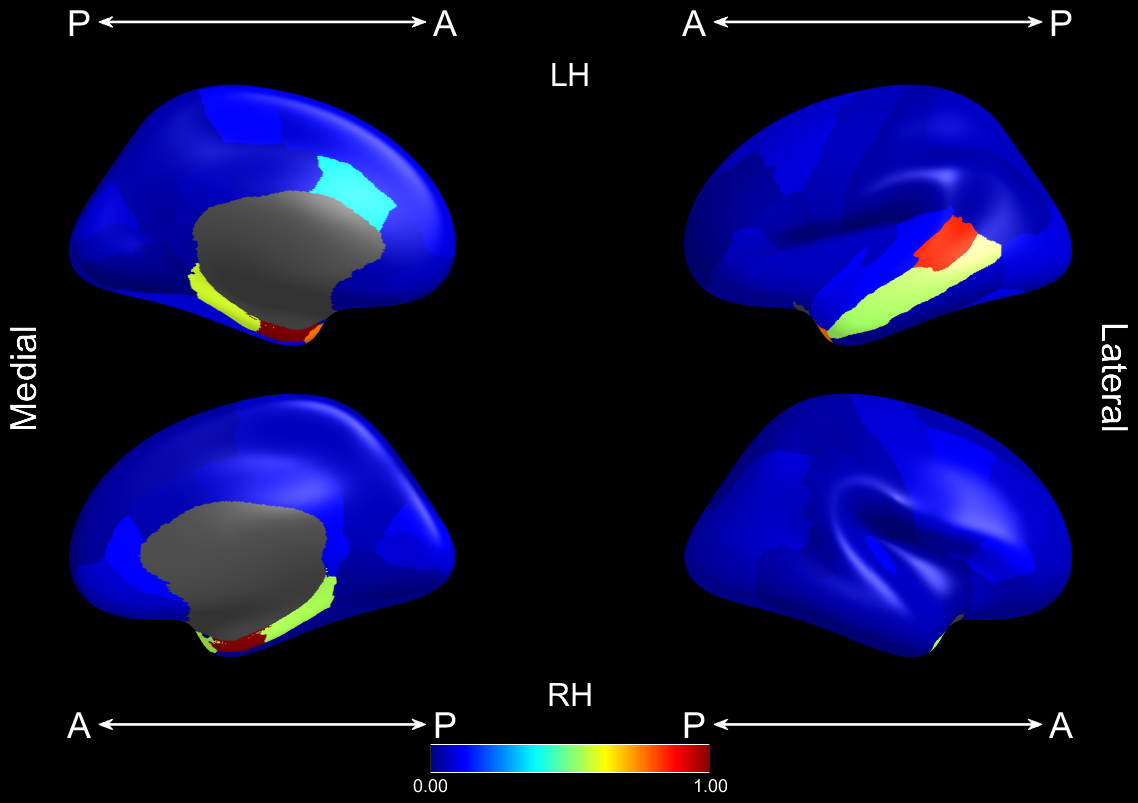


Figure S17: Feature selection probabilities per the cross-validation procedure, by-Trajectory classification scenario, “FS” feature set. Upper panel: sorted feature selection probabilities, coloured by source feature set. Lower panel: cortical thickness.


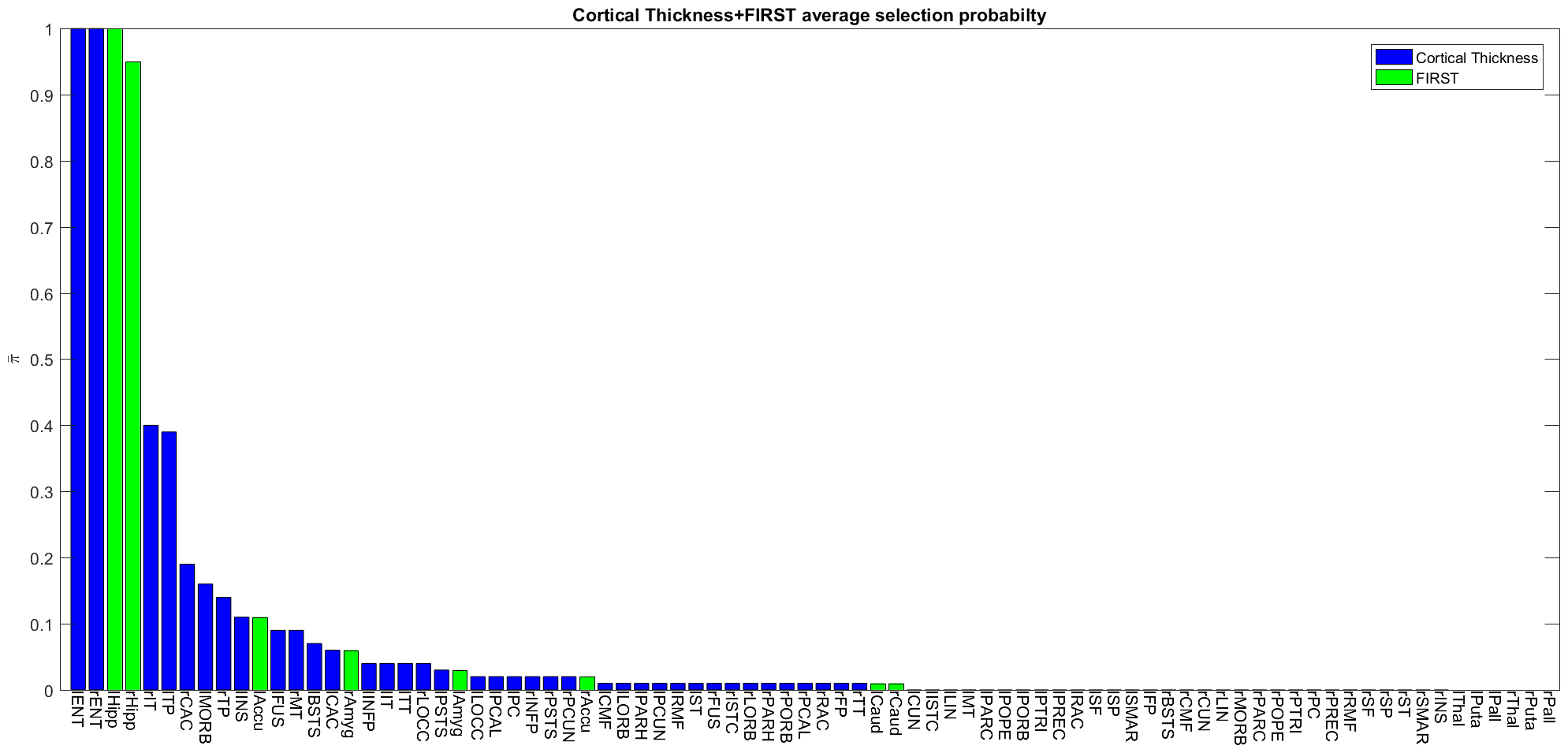

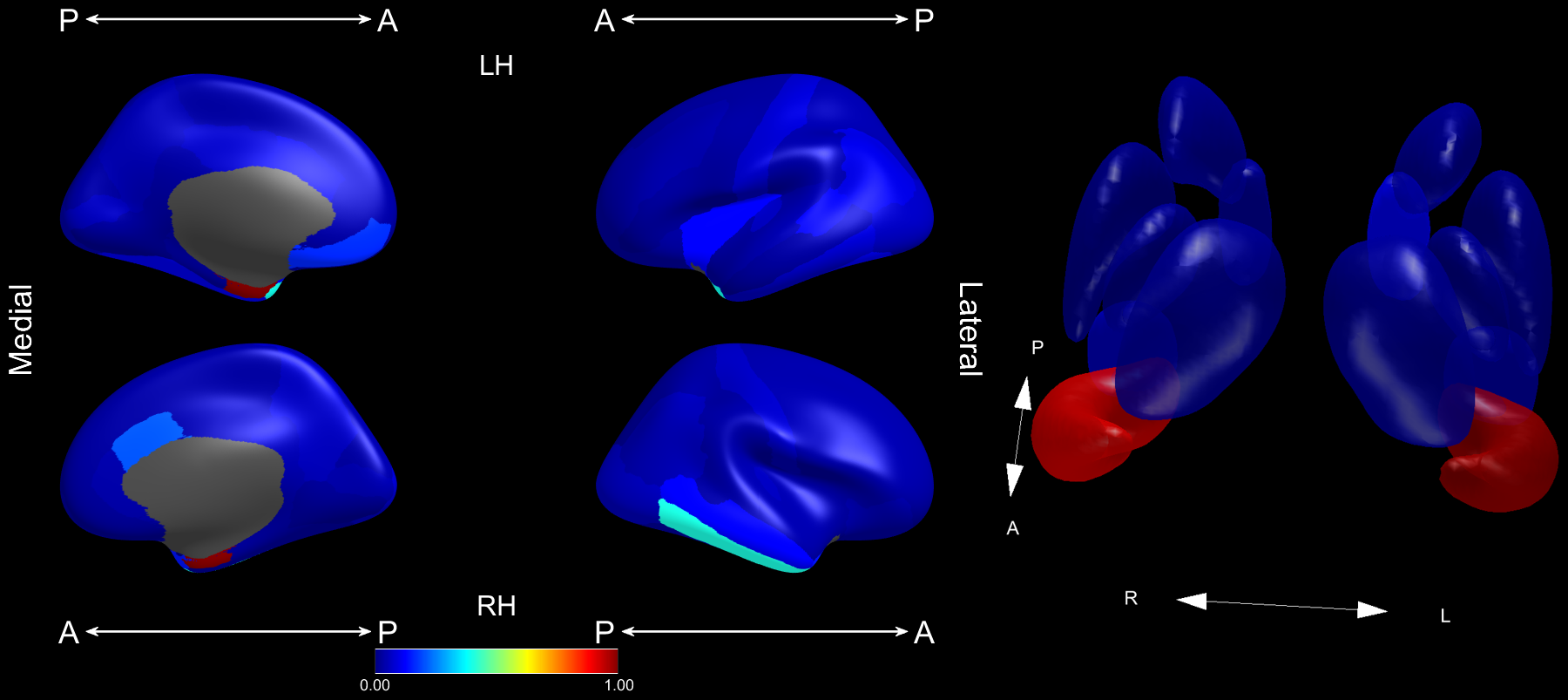


Figure S18: Feature selection probabilities per the cross-validation procedure, by-Trajectory classification scenario, “FSFIRST” feature set. Upper panel: sorted feature selection probabilities, coloured by source feature set. Lower panel: cortical thickness (left) and subcortical grey matter nuclei (right).
